# A comprehensive benchmarking for spatially resolved transcriptomics clustering methods across variable technologies, organs, and replicates

**DOI:** 10.1002/imt2.70084

**Published:** 2025-10-09

**Authors:** Renjie Chen, Yue Yao, Jingyang Qian, Xin Peng, Xin Shao, Xiaohui Fan

**Affiliations:** ^1^ Pharmaceutical Informatics Institute, College of Pharmaceutical Sciences Zhejiang University Hangzhou China; ^2^ State Key Laboratory of Chinese Medicine Modernization, Innovation Center of Yangtze River Delta Zhejiang University Jiaxing China; ^3^ Ningbo Municipal Hospital of TCM Affiliated Hospital of Zhejiang Chinese Medical University Ningbo China; ^4^ Zhejiang Key Laboratory of Precision Diagnosis and Therapy for Major Gynecological Diseases, Women's Hospital Zhejiang University School of Medicine Hangzhou China

**Keywords:** benchmarking analysis, preprocessing pipeline, spatial clustering, spatially resolved transcriptomics, systematic comparison

## Abstract

Spatial clustering is a critical step in the analysis of spatially resolved transcriptomics, serving as the foundation for downstream investigation of tissue heterogeneity. Although numerous computational tools have been developed, systematic benchmarking across different technologies, organs, and biological replicates has been limited. Here, we present a comprehensive evaluation of 14 spatial clustering methods using approximately 600 datasets, including both real‐world and simulated data with ground truth. We evaluated accuracy and applicability across diverse technologies and organs, revealing method‐specific strengths and preferences. Using simulation of adjacent tissue slices and spatial neighborhood disruptions, we further examined performance in the context of biological replicates. Furthermore, we investigated how data characteristics, spatial distribution patterns, and preprocessing pipelines influence clustering outcomes. Together, our results provide practical benchmarking guidance, enabling researchers to select appropriate spatial clustering methods tailored to specific technologies, organs, and biological replicates.

## Highlights

- We performed a comprehensive benchmarking analysis of 14 spatial clustering methods using ~600 datasets across ten technologies and eight organs.
- We provided practical recommendations for method selection for spatially resolved transcriptomics across technologies, organs, and biological replicates, involving either cell type clustering or spatial domain identification.
- We systematically assessed the influence of data characteristics and spatial patterns on clustering accuracy and offered the optimal preprocessing pipeline covering normalization, log transformation, gene selection, standardization, and dimension reduction steps for spatial clustering methods.

## Graphical abstract

Spatial clustering is a critical step in spatially resolved transcriptomics. Here, we conducted a comprehensive benchmarking analysis of 14 spatial clustering methods using ~600 datasets, including both real‐world and simulated data spanning ten technologies and eight organs. We provide practical recommendations for method selection across diverse technologies, organs, and biological replicates. Performance differences are largely explained by data characteristics, spatial patterns, and preprocessing strategies, and we establish an optimized preprocessing pipeline to enhance clustering robustness.

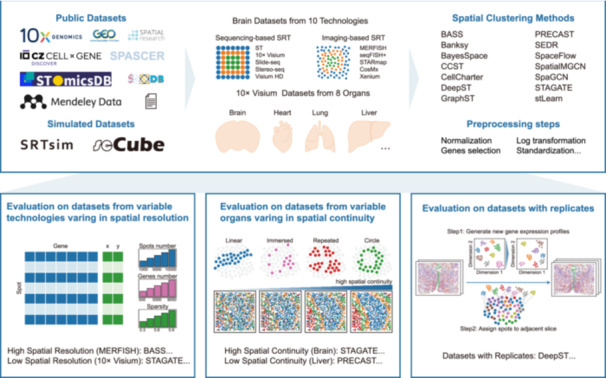

## INTRODUCTION

The advent of spatially resolved transcriptomics (SRT), which profiles gene expression while preserving spatial context, has revolutionized the study of tissue heterogeneity. SRT has been applied to investigate diverse biological processes, including the roles of distinct cell types in maintaining tissue homeostasis, the spatial dynamics of development, tumor microenvironmental organization, and tissue disarray in diseases [1, 2, 3, 4]. A critical step in analyzing the high‐dimensional data of SRT is the identification of tissue structures through spatial clustering. This foundational task underpins downstream analyses such as the detection of spatially variable genes [5], inference of spatially organized cell‐cell interactions [6, 7], spatial trajectory reconstruction [8], and the characterization of regional disease‐associated alterations [9].

Existing computational frameworks for single‐cell transcriptomics, such as Seurat [10] and Scanpy [11], rely primarily on transcriptional similarity for clustering, but do not explicitly account for spatial neighborhood information. To address this limitation, a growing number of computational methods have been developed specifically for SRT data. However, most have been evaluated on only a limited set of datasets, raising questions about their robustness and generalizability in practical applications. Choosing the most suitable tool is further complicated by the heterogeneity of platforms and organs. Broadly, classical SRT technologies fall into two categories: (1) next‐generation sequencing‐based approaches, which encode spatial coordinates with barcodes (e.g., ST [12], 10× Visium [13, 14], Slide‐seq [15, 16], Stereo‐seq [17], Visium HD [18]); and (2) imaging‐based approaches, which directly capture transcript localization (e.g., seqFISH+ [19], MERFISH [20], STARmap [21], CosMx [22], Xenium [23]). These platforms differ not only in file formats but also in spatial resolution and gene detection throughput, all of which influence the performance and applicability of clustering algorithms. Previous benchmarking studies suggest that no single method achieves optimal performance across all technologies [24, 25]. Furthermore, even within a given technology, performance can vary depending on organ‐specific architecture and the intrinsic variability of gene expression. However, comprehensive evaluation across diverse organs and biological replicates remains lacking, and the factors driving performance variability have not been systematically examined.

Here, we present a comprehensive benchmarking of 14 spatial clustering methods across multiple SRT technologies and organs using real‐world datasets with reliable ground truth. We further leveraged scCube [26], a simulator specifically designed for SRT data, to evaluate performance under scenarios involving biological replicates and a disrupted spatial neighborhood. Beyond benchmarking, we systematically examine factors that influence clustering accuracy, including data characteristics and spatial distribution patterns. Together, these analyses offer a practical framework for selecting appropriate clustering methods, thereby establishing a robust foundation for downstream SRT analysis.

## RESULTS

### Overview of datasets and spatial clustering methods for benchmarking

**Figure 1:**
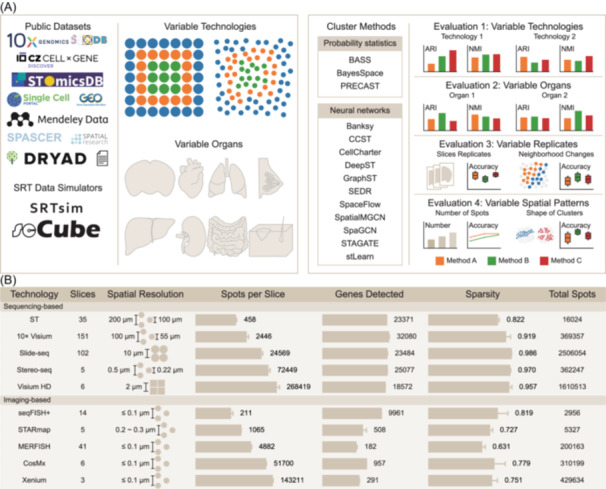
Overview of datasets and methods for benchmarking. (A) Workflow of the benchmarking study. Real datasets collected from public databases, together with simulated datasets generated with SRTsim and scCube, were used to evaluate spatial clustering methods across multiple application scenarios. Fourteen spatial clustering methods, spanning both probabilistic statistics and neural network‐based methods, were compared for accuracy across technologies, organs, biological replicates, and simulated spatial patterns. (B) Summary of real datasets used in the study. Datasets obtained from ST, 10× Visium, Slide‐seq, Stereo‐seq, Visium HD, seqFISH+, STARmap, MERFISH, CosMx, and Xenium technologies are shown, including the number of slices, spatial resolution, number of spots, genes, and sparsity per slice. Bar lengths represent the mean number of spots, and error bars indicate standard deviation.

To evaluate spatial clustering methods under diverse real‐world conditions, we assembled datasets from publicly available resources, including Cellxgene [27], STOmicsDB [28], Single Cell Portal [29], SODB [30], and SPASCER [31] (Figure 1A and Table S1). Each slice included either expert manual annotation or previously validated clustering results, which we treated as ground truth. Given the central role of reliable ground truth in benchmarking, we thoroughly reviewed their provenance and ensured that only biologically validated datasets were included (Figure S1). Collectively, these datasets span a broad spectrum of SRT platforms, including ST, 10× Visium, Slide‐seq, Stereo‐seq, Visium HD, seqFISH+, STARmap, MERFISH, CosMx, and Xenium, and cover a range of organs, such as the brain, breast, heart, kidney, liver, and lung. Together, these datasets provide a multidimensional basis for comparison across both technological platforms and biological contexts (Figure 1B).

We benchmarked 14 spatial clustering methods, encompassing both classical and recently developed approaches: BASS [32], Banksy [33], BayesSpace [34], CCST [35], CellCharter [36], DeepST [37], GraphST [38], PRECAST [39], SEDR [40], STAGATE [41], SpaGCN [42], SpaceFlow [43], SpatialMGCN [44], and stLearn [8]. To quantify clustering accuracy, we used six widely adopted evaluation metrics: Adjusted Rand Index (ARI) [45], Normalized Mutual Information (NMI) [45], Fowlkes–Mallows Index (FMI) [45], Purity [8], Homogeneity [46], and Completeness [46]. Each captures a complementary aspect of agreement between predicted clusters and ground truth. To facilitate comparison, we calculated an average rank across all metrics as an overall accuracy score to summarize performance. We additionally recorded runtime and peak memory usage to assess computational efficiency. To capture spatial continuity, we further evaluated Average Silhouette Width (ASW) [47], CHAOS [48], and Percentage of Abnormal Spots (PAS) [48], which assess the continuity of cluster assignments across adjacent spots. All methods were implemented following their tutorial and default parameters, with minor modifications as needed for compatibility across datasets. For example, BayesSpace requires a square or regular hexagonal grid as input, restricting its application to ST and 10× Visium data.

To address variability arising from biological replicates, we used scCube [26] in reference‐based simulation mode, generating synthetic slices with identical spatial distribution based on the gene expression matrix of adjacent real slices. This design enabled unbiased evaluation of method stability across biological replicates. We also examined the role of spatial neighborhood structure by merging or introducing clusters within known tissue architectures, revealing the importance of adjacency in defining organizational context.

Finally, to probe the factors underlying performance variability, we used both SRTsim [49] and scCube [26] to generate controlled simulations. These datasets varied in the number of genes and spots, and levels of sparsity, reflecting the technical diversity across SRT platforms. Tissue morphology and spatial distribution patterns, such as the number and shape of clusters, were also systematically varied to assess sensitivity to biological complexity. In parallel, we assessed the effects of preprocessing pipelines and multi‐slice integration strategies, demonstrating that analytical choices can substantially influence clustering accuracy.

### Performance comparison with SRT datasets across technologies

**Figure 2:**
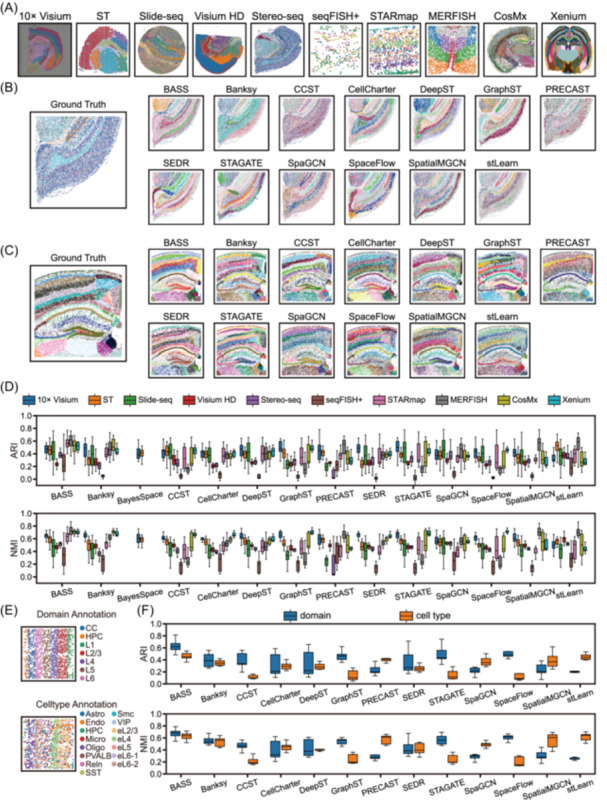
Performance comparison with SRT datasets across technologies. (A) Ground‐truth annotations for one representative slice from each technology. (B) Ground‐truth and clustering results from all methods on slice *Mouse_Hemi_Brain_Cell_Bin_Data_Whole_sub2* (Stereo‐seq). (C) Ground‐truth and clustering results from all methods on slice *Mouse_Brain_MultiSection 1* (Xenium). (D) Box plots show clustering accuracy, measured by Adjusted Rand Index (ARI) and Normalized Mutual Information (NMI), on brain datasets across different technologies. Centerline: median; box limits: upper and lower quartiles; whiskers: 1.5× interquartile range. (E) Ground‐truth annotations for slice *20180505_BY3_1kgenes* (STARmap), shown at both spatial domain and cell type levels. (F) Box plots show clustering accuracy, measured by ARI and NMI, on datasets with different levels of ground‐truth annotations. Centerline: median; box limits: upper and lower quartiles; whiskers: 1.5× interquartile range. SRT, spatially resolved transcriptomics.

To compare clustering performance across technologies, we analyzed datasets from 10 classic and widely used platforms: ST, 10× Visium, Slide‐seq, Stereo‐seq, Visium HD, seqFISH+, STARmap, MERFISH, CosMx, and Xenium (Figure 2A). For each platform, we present representative clustering results from a typical tissue slice, highlighting Stereo‐seq and Xenium specifically as examples of sequencing‐based and imaging‐based methods, respectively (Figure 2B,C, Figures S2–S7). These technologies differ markedly in spatial resolution, number of spots, and transcript throughput, which in turn influence clustering complexity and accuracy.

We first benchmarked methods with widely used metrics ARI and NMI, which provide overall measures of concordance with ground truth (Figure 2D). Additional evaluation metrics were included to balance their complementary strengths and limitations (Figure S8). For instance, the Purity score emphasizes agreement with the dominant ground‐truth label in the clustering results, but can be biased by cluster number and imbalance. In contrast, Completeness rewards methods that group all the same ground‐truth labels, even at the cost of internal heterogeneity. This trade‐off was evident with stLearn, which achieved high Completeness but low Homogeneity on ST datasets. Similarly, Banksy on Slide‐seq data ranked poorly in Purity (10th) and ARI (7th), indicating weak local concordance, but performed better in Completeness (4th), suggesting preservation of the global structure of cell types. To integrate across metrics, we derived a comprehensive accuracy score based on the median rank across all measures (Figure S9).

Method performance varied by platform. On 10× Visium brain datasets, STAGATE showed the highest accuracy (e.g., median ARI = 0.498), followed by GraphST (0.483). On ST datasets, BASS (0.461) and BayesSpace (0.415) were most effective. For Slide‐seq, STAGATE (0.490) and SpaGCN (0.458) outperformed other methods, followed by BASS and CCST. By contrast, performance declined substantially on seqFISH+ datasets, with PRECAST showing the best, though modest, accuracy (0.228). Notably, Banksy demonstrated optimal accuracy on CosMx datasets, which typically contain large spot numbers. Assessing stability across technologies, SEDR, SpaGCN, and DeepST consistently exhibited narrow interquartile ranges for ARI and NMI, suggesting robust though not always top‐ranked performance (Figure S10). However, computational costs varied sharply across technologies: BASS and CCST, for instance, incurred high runtime and memory usage on Stereo‐seq or Xenium datasets, which can be a limiting factor in their application on large datasets (Figure S11).

The spatial resolution of SRT platforms dictates the nature of clustering tasks. ST and 10× Visium aggregate transcripts from multiple cells per spot, constraining annotations to spatial domains. In contrast, high‐resolution platforms such as Stereo‐seq and Xenium enable single‐cell resolution, where cell‐type clustering becomes important. To explore this, we analyzed a subset of STARmap and MERFISH datasets annotated with both cell types and spatial domains. For example, in the Mouse Visual Cortex STARmap data set, spots were classified into 15 cell types based on marker gene expression and manually assigned into seven spatial layers (Figure 2E). While spatial layers displayed clear stratification, individual cell types spanned multiple layers, highlighting the added complexity of cell‐type clustering. Performance indeed varied by clustering tasks, even within the same data set. Specifically, SpaceFlow, GraphST, STAGATE, and CCST were particularly effective for spatial domain identification, whereas stLearn, PRECAST, SpatialMGCN, and SpaGCN were better suited for cell type identification. BASS outperformed most methods across both tasks, and Banksy demonstrated relatively balanced results (Figure 2F).

We further investigated spatial niches, defined as the local composition of cell types and often conceptualized as analogous to spatial domains (Figure S12). Spatial niche detection is particularly relevant in complex tissues such as tumors or immune‐rich environments. Here, Banksy, SpaceFlow, BASS, CCST, and CellCharter showed superior performance. Interestingly, some methods originally designed for spatial domain identification (e.g, SpaceFlow and CCST) also demonstrated reasonable ability in spatial niche detection. However, accuracy for spatial niche detection was consistently lower than for cell‐type clustering across all other methods, underscoring the greater challenge of modeling the local cellular microenvironments.

### Performance comparison with SRT datasets across organs

**Figure 3:**
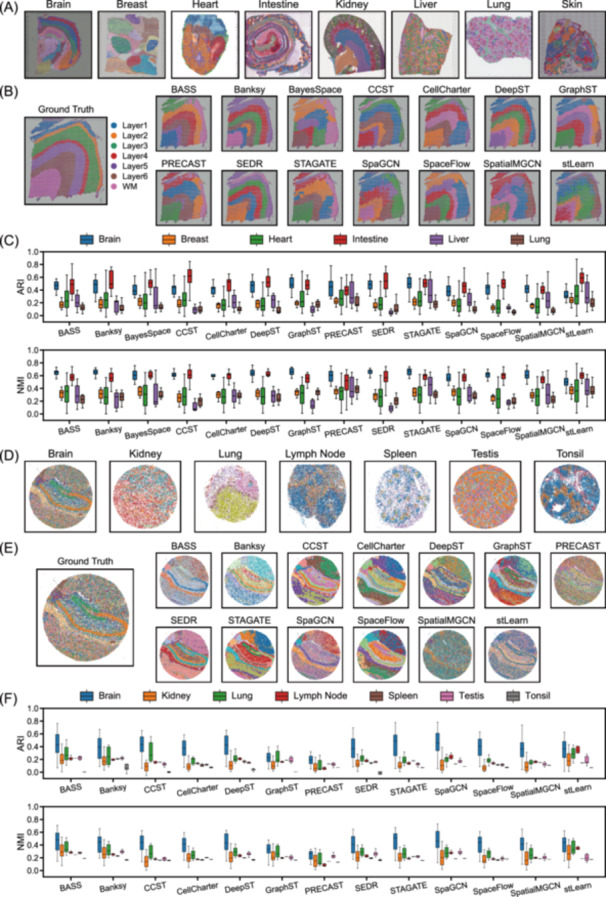
Performance comparison with SRT datasets across organs. (A) Ground‐truth annotations for representative slices from each organ in the 10× Visium datasets. (B) Ground‐truth annotations and clustering results from all methods on slice *151676* of the DLPFC 10× Visium data set. (C) Box plots compare methods on all 10× Visium datasets of variable organs with Adjusted Rand Index (ARI) and Normalized Mutual Information (NMI). Centerline: median; box limits: upper and lower quartiles; whiskers: 1.5× interquartile range. Results of kidney and skin datasets are presented in Figure S16 due to lower confidence in the ground truth. (D) Ground‐truth annotations for representative slices from each organ in the Slide‐seq datasets. (E) Ground truth and clustering result from each method on a representative Hippocampus Slide‐seq slice. (F) Comparison of methods across all Slide‐seq datasets from different organs, with clustering accuracy measured by ARI and NMI. Centerline: median; box limits: upper and lower quartiles; whiskers: 1.5× interquartile range. SRT, spatially resolved transcriptomics.

To assess how organ‐specific architecture influences clustering accuracy, we first analyzed datasets generated with the widely used 10× Visium platform, spanning brain, breast, heart, intestine, liver, lung, kidney, and skin (Figure 3A). Among these, the dorsolateral prefrontal cortex (DLPFC) data set [50], containing 12 slices from three donors, is commonly used to evaluate the performance of new methods. In slice *151676*, for example, 33,538 genes were detected across 3431 spots, annotated into six cortical and white matter regions. Across methods, clustering recapitulated aspects of cortical layering to varying extents (Figure 3B). Visual inspection revealed marked differences in spatial organization between organs, reflecting inherent biological variability: the coronal section of the brain showed well‐defined linear layered structures, while the liver displayed repetitive, regionally clustered domains corresponding to portal triads and central veins (Figure S13). To quantify such patterns, we calculated spatial continuity metrics (PAS, CHAOS, and ASW), which capture the spatial adjacency of spots sharing the same ground‐truth labels (Figure S14).

Based on the overall accuracy score, the clustering accuracy of methods on different organs is summarized (Figure 3C, Figures S15–S17). In brain slices, STAGATE (median ARI = 0.498) and GraphST (0.483) achieved the highest accuracy in identifying domains. In intestine, CCST (0.619), DeepST (0.521), and CellCharter (0.527) accurately delineated layers such as the mucosa, submucosa, and muscularis. For breast tissue, stLearn (0.236), PRECAST (0.220), and BayesSpace (0.225) were top performers, while for heart, stLearn (0.305) and PRECAST (0.265) showed superior results.

From a spatial continuity perspective (Figure S18), SEDR, SpaceFlow, and BASS ranked highest (with top‐ranked PAS, CHAOS, and ASW scores), suggesting suitability for organs with continuous structures. However, spatial continuity metrics calculated with ground truth differed by organ. In organs with intrinsically low continuity, such as liver and lung, methods emphasizing continuous clusters produced results inconsistent with ground truth. This may explain why the accuracy of these methods was not optimal in some cases, highlighting the influence of cluster geometry and distribution characteristics on performance.

Since 10× Visium datasets contained a moderate number of spots ranging from 1290 to 3492, differences in computational cost were not driven by data set size. Instead, algorithmic design dictated runtimes and memory usage among different methods (Figure S19). Our results showed that, regardless of the organs, Bayesian inference‐based methods such as BASS and BayesSpace consistently incurred higher runtime and memory consumption. For methods integrating gene expression profiles and H&E‐stained histological images, like stLearn and DeepST, require additional resources for histology feature extraction.

We next benchmarked methods on Slide‐seq datasets, which covered brain, kidney, lung, lymph node, testis, spleen, and tonsil organs (Figure 3D). At near single‐cell resolution, Slide‐seq captures more complex and disordered cell‐type distribution patterns (Figure 3E). Compared with 10× Visium datasets, BASS achieved consistently strong performance on most organs. Specifically, in brain slices, STAGATE, SpaGCN, and BASS ranked highest, while BASS and stLearn excelled in kidney and lung. Banksy scored the highest in the testis datasets. Notably, some methods show organ‐specific strengths: CCST ranked fourth on brain data, DeepST ranked fourth on lymph node and spleen, and SpaceFlow ranked fifth on tonsil (Figure 3F). Spatial continuity was generally low across Slide‐seq datasets and relatively similar across different organs (Figure S20), as neighboring spots often represented distinct cell types. This lower spatial continuity challenged methods that rely heavily on neighborhood similarity, contributing to the overall reduced accuracy. By contrast, methods such as BASS and Banksy, which employ flexible spatial modeling and hierarchical inference, adapted better to the high sparsity and complexity of Slide‐seq datasets.

Together, these results underscore that clustering accuracy is shaped jointly by the intrinsic spatial organization of organs and the design assumptions of computational methods. Methods that impose strong spatial continuity perform well in layered tissues but may misclassify heterogeneous structures, where flexible approaches are resilient to complex, cell‐type‐rich environments.

### Performance comparison with SRT datasets across biological replicates

**Figure 4:**
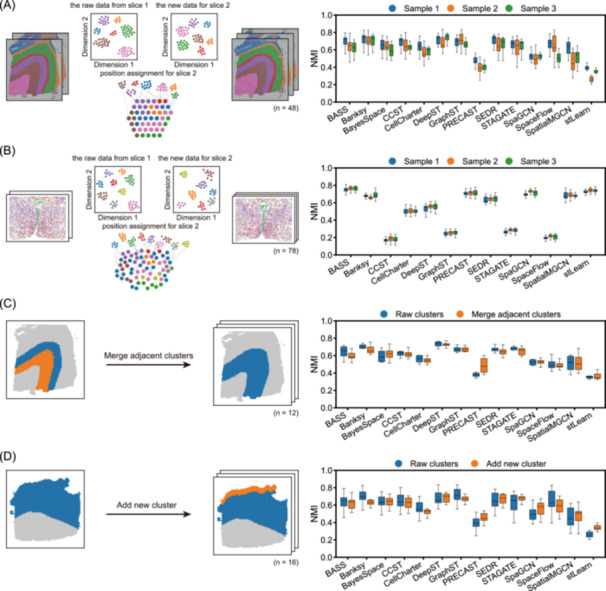
Performance comparison with SRT datasets across biological replicates. (A) Simulation of variable replicates based on DLPFC 10× Visium datasets. Box plot shows Normalized Mutual Information (NMI) scores on simulated datasets. (B) Simulation of variable replicates based on the hypothalamus MERFISH data set. Box plot shows NMI scores on simulated datasets. (C) Design of simulation for neighborhood‐changing replicates by merging adjacent clusters. Box plot shows NMI scores on simulated datasets. (D) Design of simulation for neighborhood‐changing replicates by adding new clusters. Box plot shows NMI scores on simulated datasets. The box represents the interquartile range, the horizontal line inside the box indicates the median, and the whiskers extend to 1.5× interquartile range. SRT, spatially resolved transcriptomics.

SRT data often vary between tissue sections, even among adjacent slices from the same biological sample, due to batch effects or subtle spatial discrepancies. To benchmark clustering robustness under such conditions, we used scCube [26] to simulate biological replicates, allowing for a realistic comparison of method performance. To be specific, scCube learns cluster‐specific gene expression characteristics from one slice and generates corresponding spots in adjacent slices, thereby creating replicate datasets without batch effects. We used the DLPFC 10× Visium data set and the hypothalamus MERFISH data set as references, both of which contain multiple slices from the same tissue with broadly consistent domains but slight boundary shifts. Notably, most methods achieved comparable NMI scores on simulated and real datasets, particularly in MERFISH data, validating our simulation framework in capturing key biological variability (Figure S21). On 10× Visium data, DeepST, Banksy, SEDR, GraphST, and STAGATE achieved the highest accuracy (Figure 4A), whereas in MERFISH datasets, BASS, stLearn, SpaGCN, and PRECAST outperformed others (Figure 4B). Importantly, performance varied across different sections, highlighting the difficulty of achieving stable accuracy across multiple samples. This variability likely arises from differences in algorithmic assumptions. For example, methods that rely on strong spatial smoothness or heavily depend on graph construction are especially sensitive to small boundary shifts.

Additionally, in spatial clustering, it is important to consider both gene expression patterns and spatial neighborhood relationships. However, spatial structure may be disrupted by tissue sectioning, sample preservation, or biological processes such as tumor growth or disease progression. During tissue development, spatially adjacent domains may also exhibit dependency relationships. To further test robustness, we simulated spatial neighborhood disruptions with DLPFC slice *151673*, merging two adjacent clusters to reduce the original seven‐layer structure to six layers (Figure 4C). Although this should, in principle, simplify the clustering task with fewer cluster numbers, accuracy decreased for most methods, including BASS, Banksy, and SEDR. Artificial homogenization weakened expression contrasts and altered boundaries, misleading algorithms that rely on spatial graphs or neighborhood information. Conversely, DeepST, SEDR, GraphST, and STAGATE maintained relatively high accuracy.

Moreover, we also tested the effect of adding new clusters into slices where some structures were originally missing. For instance, in DLPFC slice *151669*, which lacks Layer 1 and 2, we inserted Layer 2 profiles sampled from other tissue sections and positioned them adjacent to Layer 3 (Figure 4D). Accuracy again declined in several methods, likely because the added cluster disrupted natural spatial continuity and introduced artificial boundaries. Increasing local structural complexity also confused some algorithms. Nonetheless, DeepST, STAGATE, and SEDR preserved relatively high NMI scores, indicating robustness to disruptions in spatial structure.

Collectively, these data demonstrate that clustering accuracy across replicates is influenced not only by biological and technical variations but also by algorithmic design. Methods such as DeepST, SEDR, and STAGATE, which flexibly integrate spatial and transcriptomic information, exhibit greater resilience to shifts in domain boundaries and perturbations of spatial neighborhoods.

### Performance comparison with SRT datasets from variable spatial patterns

Given the variability observed across technologies and organs, we next explored how intrinsic spatial patterns affect clustering accuracy using simulated datasets.

**Figure 5:**
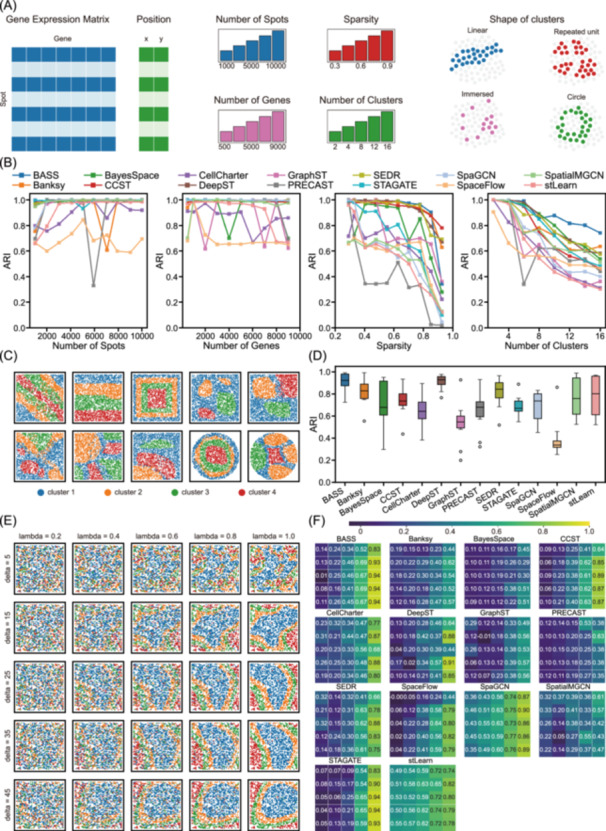
Performance comparison with SRT datasets from variable spatial patterns. (A) Design of simulation for variable spatial patterns. (B) Line plots show Adjusted Rand Index (ARI) changes with different spot numbers, gene numbers, sparsity, and cluster numbers. (C) Ground‐truth annotations of the custom‐designed shape of clusters. (D) Box plot of the ARI score compares methods on the shape of clusters. Centerline: median; box limits: upper and lower quartiles; whiskers: 1.5× interquartile range. (E) Ground‐truth annotations of simulated datasets generated with varying λ (lambda) and δ (delta) parameters. Larger λ values produce clearer spatial patterns, while larger δ values yield greater connectivity. (F) Heatmap of ARI scores for each method across simulated datasets with varying λ and δ parameters. SRT, spatially resolved transcriptomics.

We first investigated sensitivity to data characteristics with SRTsim (Figure 5A). Higher spatial resolution increases the number of sampling spots within a given area but also introduces technical limitations in transcript capture, reflected as differences in matrix dimensions and sparsity. As sparsity increased, clustering accuracy declined across most methods (Figure 5B). BASS, Banksy, and DeepST were particularly robust, maintaining ARI > 0.9 even under high sparsity. In contrast, SpaGCN, STAGATE, and PRECAST were highly sensitive, with PRECAST dropping from near‐perfect ARI at low sparsity to 0.019 at 92.5% sparsity, consistent with its poor performance on sparse Slide‐seq and Stereo‐seq datasets. By comparison, the number of spots or genes had little effect when sparsity was low, where ARI scores remained in the range of 0.9 to 1. For methods such as PRECAST, SpaGCN, and stLearn improved modestly when spot counts were less than 3000. Besides, the number of spots was a key factor influencing the runtime and memory usage of the methods, which was consistent with real‐data benchmarks (Figure S22). Thus, sparsity emerged as the dominant factor shaping accuracy, whereas spot number mainly affected computational efficiency.

As spatial resolution advances toward single‐cell and subcellular levels, clustering shifts from coarse domain identification to fine‐grained cell‐type classification, which tends to be larger in the number of spatial clusters and shows intermingled and discontinuous distribution features. To simulate this, we varied the number of target clusters. Accuracy decreased as cluster numbers increased, with ARI scores falling from 1 to 0.4–0.8 and regression coefficients consistently negative (−0.3 to −0.8) (Figures 5B, S23). This demonstrates the inherent difficulty of resolving numerous clusters.

We next explored the impact of cluster geometry, as various methods show performance differences in organs with different organizational morphology and regional distribution characteristics. Using simulated datasets with linear, circular, immersed, and repetitive shapes (Figure 5C), we observed that DeepST, BASS, and SEDR were the most stable methods across cluster shapes. Almost all methods accurately reconstructed linear structures, but performance diverged on more complex shapes (Figure 5D). These results suggest that different methods have structural preferences in recognizing specific morphologies.

To test performance under more complex spatial organizations, such as immune infiltration patterns in the tumor microenvironment, we further applied scCube to simulate data under parametric control. Two parameters were adjusted: lambda (λ), which controls the fuzziness of domain boundaries, and delta (δ), which controls connectivity between spatial regions. Increasing λ yielded clearer spatial patterns, while higher δ produced spatial patterns with greater connectivity. We generated 25 datasets by varying these parameters on the same scRNA‐seq reference (Figure 5E). Ground‐truth spatial continuity, as measured by PAS and CHAOS, ranged from 0.11 to 0.56 and 0.07 to 0.10, respectively, across simulated datasets. Clustering accuracy strongly correlated with λ in most methods, including BASS, CCST, CellCharter, SpaceFlow, and STAGATE (Pearson *r* > 0.9), with ARI values increasing from 0.1 to >0.9 as λ increased (Figures 5F, S24). SpaGCN and stLearn did not reach the highest accuracy at large λ, but maintained relatively good performance at low λ or δ, with ARI scores between 0.35 and 0.55, which may explain their ability to handle datasets with high mixing or disordered domains.

Finally, given the importance of preprocessing in real‐world applications, we systematically tested the effects of normalization, log transformation, gene selection, standardization, and dimension reduction on clustering accuracy. We identified the top eight configurations based on ARI scores and summarized the optimal pipeline for each method (Figures S25, 26). Importantly, different methods exhibited varying sensitivity to preprocessing steps. For instance, DeepST was strongly affected by the number of principal components used for principal component analysis (PCA), while GraphST remained stable across settings (Figures S27, 28). BASS and STAGATE achieved peak accuracy using default preprocessing, whereas other methods benefited from customized pipelines, suggesting that well‐designed preprocessing pipelines can enhance robustness across different scenarios. Across methods, normalization was indispensable, and selecting 3000 spatially variable genes using Spark‐X instead of standard highly variable genes (HVG) selection improved clustering performance in most methods. The impact of log transformation and standardization varied considerably, as both operations reshape the variance structure in the data. For PCA‐based methods (e.g., DeepST, SEDR, CCST), the default use of 200 components was often suboptimal; in several cases, reducing the number of principal components to 20 suppressed noise and improved accuracy. The most effective general pipeline included: (1) library‐size normalization, (2) log transformation, (3) selection of 3000 spatially variable genes, (4) no standardization, and (5) for methods requiring dimensionality reduction, using the top 20 principal components. We also compared integrated versus single‐slice analysis. Although integration facilitated visualization of cross‐slice continuity, it yielded no statistically significant accuracy improvement in most methods (Figure S29).

## DISCUSSION

In this study, we systematically benchmarked 14 spatial clustering methods across diverse SRT datasets, spanning different technologies, organs, and biological replicates. We further evaluated the effects of spatial patterns and the preprocessing pipeline on clustering accuracy. Our large‐scale analysis revealed that no single method consistently outperforms others across all scenarios, underscoring that each method has strengths in a specific context. To guide practical applications, we summarized method performance and provided recommendations (Table 1 and Figures S30, 31). In summary, on 10× Visium datasets across organs, STAGATE, DeepST, and SEDR were generally optimal, especially for tissues with strong spatial continuity such as the brain cortex, whereas PRECAST and stLearn performed better on organs with lower continuity, such as the lung and liver. BASS emerged as the most reliable choice for high‐resolution datasets like Slide‐seq. For replicate slices, methods such as DeepST, Banksy, SEDR, GraphST, and STAGATE achieved consistently high accuracy and robustness, even under spatial neighborhood disruptions (Table S2).

**Table 1:** Summary of ARI and NMI scores for methods across all scenarios.

		Metric	BASS	Banksy	BayesSpace	CCST	CellCharter	DeepST	GraphST	PRECAST	SEDR	STAGATE	SpaGCN	SpaceFlow	SpatialMGCN	stLearn
Variable Organs	Brain	ARI	0.474	0.489*	0.386	0.443	0.425	0.476	0.483	0.452	0.485*	0.498*	0.377	0.440	0.423	0.306
		NMI	0.655	0.655	0.608	0.611	0.593	0.637	0.661*	0.606	0.679*	0.671*	0.556	0.622	0.603	0.500
	Breast	ARI	0.166	0.170	0.225*	0.169	0.170	0.189	0.195	0.220*	0.138	0.214	0.199	0.124	0.167	0.236*
		NMI	0.333	0.317	0.348	0.255	0.304	0.326	0.348	0.361*	0.296	0.336	0.353*	0.273	0.292	0.375*
	Heart	ARI	0.229	0.242*	0.197	0.240	0.163	0.151	0.217	0.265*	0.169	0.224	0.149	0.113	0.133	0.305*
		NMI	0.354*	0.265	0.310	0.271	0.267	0.269	0.328	0.356*	0.269	0.299	0.271	0.236	0.279	0.359*
	Intestine	ARI	0.488	0.493	0.512	0.619*	0.527	0.521	0.476	0.391	0.540*	0.505	0.465	0.501	0.396	0.590*
		NMI	0.574	0.560	0.599	0.609*	0.614*	0.617*	0.560	0.521	0.568	0.602	0.575	0.608	0.545	0.604
	Liver	ARI	0.200	0.218	0.159	0.095	0.203	0.246	0.089	0.277*	0.042	0.317*	0.204	0.114	0.229	0.279*
		NMI	0.278	0.269	0.189	0.081	0.282	0.285	0.126	0.352*	0.088	0.458*	0.286	0.174	0.298	0.363*
	Lung	ARI	0.119	0.095	0.128	0.087	0.096	0.068	0.171	0.172*	0.087	0.178*	0.096	0.048	0.068	0.182*
		NMI	0.228	0.267	0.280	0.209	0.275	0.229	0.348*	0.396*	0.212	0.301	0.261	0.179	0.216	0.346*
Variable Technologies	ST	ARI	0.461*	0.317	0.415	0.436*	0.399	0.402	0.411	0.421	0.230	0.390	0.365	0.314	0.334	0.530*
		NMI	0.610*	0.521	0.595*	0.563	0.549	0.557	0.518	0.590*	0.475	0.576	0.549	0.502	0.539	0.570
	10× Visium	ARI	0.474	0.489*	0.386	0.443	0.425	0.476	0.483	0.452	0.485*	0.498*	0.377	0.440	0.423	0.306
		NMI	0.655	0.655	0.608	0.611	0.593	0.637	0.661*	0.606	0.679*	0.671*	0.556	0.622	0.603	0.500
	Slide‐seq	ARI	0.429	0.392	N/A	0.433*	0.376	0.426	0.230	0.194	0.369	0.490*	0.458*	0.405	0.363	0.368
		NMI	0.480*	0.447	N/A	0.441	0.417	0.432	0.335	0.269	0.410	0.513*	0.458*	0.441	0.413	0.439
	Stereo‐seq	ARI	0.376*	0.214	N/A	0.242	0.212	0.275	0.298	0.021	0.258	0.326	0.362*	0.265	0.288	0.341*
		NMI	0.523*	0.365	N/A	0.403	0.387	0.433	0.436	0.043	0.436	0.475	0.534*	0.426	0.439	0.518*
	Visium HD	ARI	0.208	0.382*	N/A	0.525*	0.210	0.289	0.285	0.294	0.328	0.290	0.304	0.198	0.267	0.391*
		NMI	0.414	0.490*	N/A	0.429	0.376	0.458	0.433	0.392	0.469*	0.417	0.460	0.353	0.463	0.534*
	seqFISH+	ARI	0.209*	0.050	N/A	0.052	0.080	0.031	0.046	0.228*	0.008	0.015	0.083	0.020	0.038	0.175*
		NMI	0.346*	0.270	N/A	0.186	0.283	0.201	0.219	0.490*	0.148	0.140	0.250	0.167	0.155	0.290*
	STARmap	ARI	0.564*	0.385	N/A	0.321	0.363	0.401*	0.344	0.378	0.269	0.298	0.354	0.262	0.304	0.396*
		NMI	0.638*	0.533*	N/A	0.430	0.499	0.466	0.443	0.474	0.386	0.426	0.425	0.414	0.375	0.510*
	MERFISH	ARI	0.621*	0.463	N/A	0.160	0.386	0.403	0.149	0.550	0.379	0.170	0.474	0.157	0.573*	0.590*
		NMI	0.721*	0.623	N/A	0.224	0.524	0.475	0.227	0.671	0.537	0.234	0.559	0.188	0.706*	0.699*
	CosMx	ARI	0.434*	0.419*	N/A	0.187	0.219	0.322	0.230	0.254	0.328*	0.168	0.307	0.174	0.295	0.266
		NMI	0.621*	0.620*	N/A	0.327	0.416	0.491	0.375	0.460	0.520*	0.327	0.465	0.325	0.514	0.491
	Xenium	ARI	0.477*	0.415*	N/A	0.200	0.186	0.352	0.230	0.269	0.334	0.210	0.311	0.185	0.319	0.388*
		NMI	0.632*	0.633*	N/A	0.375	0.449	0.540	0.312	0.532	0.578	0.428	0.548	0.384	0.585	0.591*
Variable Replicates	10× Visium Replicates	ARI	0.532	0.577	0.509	0.516	0.462	0.616*	0.576	0.314	0.599*	0.595*	0.416	0.462	0.419	0.222
		NMI	0.668	0.714*	0.630	0.645	0.606	0.731*	0.686	0.425	0.697*	0.674	0.524	0.601	0.551	0.355
	MERFISH Replicates	ARI	0.689*	0.530	N/A	0.122	0.369	0.520	0.171	0.589	0.522	0.189	0.640*	0.153	0.524	0.641*
		NMI	0.761*	0.671	N/A	0.175	0.503	0.556	0.257	0.714	0.643	0.279	0.718*	0.206	0.680	0.736*
	Merge Cluster	ARI	0.423	0.527	0.487	0.487	0.388	0.619*	0.548*	0.384	0.520	0.552*	0.401	0.332	0.351	0.210
		NMI	0.581	0.652	0.621	0.615	0.547	0.727*	0.671*	0.476	0.644	0.663*	0.530	0.477	0.502	0.344
	Add Cluster	ARI	0.452	0.428	0.488	0.446	0.342	0.564*	0.512	0.344	0.513*	0.523*	0.457	0.384	0.345	0.208
		NMI	0.638	0.630	0.646	0.631	0.532	0.700*	0.670	0.447	0.682*	0.687*	0.583	0.595	0.509	0.336

Abbreviations: ARI, Adjusted Rand Index; NMI, Normalized Mutual Information.

^(a)^ For each scenario, the top 3 methods with the highest score are marked with an asterisk (*).

^(b)^ Cases where the BayesSpace method is not applicable are indicated with empty cells (N/A).

Previous works have noted that clustering performance declines when analyzing small, discontinuous tissue regions or complex datasets [24, 25]. However, the factors underlying these limitations remained underexplored. Our analysis identified two major contributors: (1) intrinsic data characteristics and (2) spatial distribution patterns. Sparsity emerged as a critical determinant of performance. High sparsity, a common feature of SRT data, reduced clustering accuracy for most methods, whereas algorithms more robust to sparsity (e.g., BASS, Banksy, DeepST) maintained stable accuracy. Furthermore, increasing spatial resolution and throughput also generated large‐scale datasets requiring substantial computational resources, highlighting the need to balance accuracy with practice efficiency.

A second factor lies in organ‐specific architecture, which differs in continuity of spatial organization and spatial distribution of biologically meaningful clusters. Some tissues, such as the cerebral cortex, exhibit highly continuous, layered organization, while others (e.g., liver and lung) display more irregular structure with low continuity. Many methods fail to account for such heterogeneity, limiting their generalizability. Indeed, several new approaches have been designed for specific structural contexts or to capture fine‐scale structures [51, 52], reflecting the growing recognition that tissue‐specific features may require tailored modeling strategies.

Spatial clustering also differs fundamentally from single‐cell clustering, which primarily focuses on transcriptional heterogeneity [53, 54]. Spatial clustering must integrate both gene expression and the spatial relationship. Our benchmarking revealed that disruptions in spatial organization, such as altered boundaries or missing neighborhoods, substantially affect clustering accuracy. Most methods define spatial neighborhoods through fixed Euclidean radii or nearest neighbor graphs, with parameters set empirically to balance spatial and expression features. Many methods also assume spatial smoothness, which encourages neighboring spots to belong to the same cluster. While this facilitates spatial domain identification, it can impair performance on cell‐type clustering, especially in high‐resolution datasets [55, 56]. Accurate clustering, therefore, requires not only tuning the target number of clusters but also incorporating prior knowledge of tissue architecture. Models such as BASS [32], which employs hierarchical inference, and Banksy [33], which allows task‐specific weighting of spatial versus transcriptomic features, exemplify strategies that adapt flexibly across spatial technologies and clustering tasks.

In addition, preprocessing pipelines also play a critical role in determining clustering performance [57]. We identified optimal parameter combinations for each method and key preprocessing steps relevant to clustering robustness. Our analysis showed that the default pipeline used by many methods was often suboptimal, and that gene selection, normalization, and dimensionality reduction strongly influenced clustering accuracy. Therefore, we encourage users to refer to our recommended optimal pipelines and adjust key steps according to data characteristics in practice.

Integrating additional information, such as spatial multi‐omics and H&E staining images, may provide another promising direction. Several methods (SpaGCN, DeepST, stLearn) already incorporate H&E staining images, although without substantial performance gain in our benchmarks, suggesting that more optimized strategies for multimodal integration are likely required to fully leverage tissue morphology. Multi‐omics integration may offer greater benefits. For example, SpatialGlue [58] combines spatial multi‐omics data to improve spatial domain identification, suggesting that incorporating complementary modalities could enhance clustering accuracy and biological interpretability.

Our study has limitations. First, due to inherent differences in model design and technical constraints, we focused on benchmarking clustering accuracy rather than inferring causality between specific data features and algorithmic performance. Second, spatial clustering is only one application of the methods. Many also support cell‐type deconvolution [38], slice integration [32, 42], and pseudo‐time reconstruction [8], expanding their utility beyond clustering. Finally, our analysis did not encompass the full landscape of available methods, many of which include unique optimizations that warrant future exploration.

## CONCLUSION

In this study, we conducted a comprehensive benchmark of 14 spatial clustering methods across diverse SRT datasets covering multiple technologies, organs, and biological replicates. Using both real and simulated data, we demonstrated that biological replicates and spatial neighborhood disruptions substantially affect clustering accuracy. Performance variability was largely driven by intrinsic data set characteristics and methodological design choices, with gene expression sparsity and cluster distribution emerging as critical determinants. Preprocessing strategies also exerted variations in performance, and we identified key steps that shape clustering robustness. Collectively, these findings delineate the strengths and limitations of current spatial clustering approaches, provide actionable guidance for their application, and establish a framework for building a more reliable and consistent analytical pipeline in SRT.

## METHODS

### Datasets

The human DLPFC 10× Visium data set [50] was downloaded from http://spatial.libd.org/spatialLIBD/, containing 12 tissue slices from 3 donors. The Mouse Brain Section Coronal 10× Visium data set [59] was downloaded from https://support.10xgenomics.com/spatial-gene-expression/datasets, containing 1 tissue slice. The mouse brain 10× Visium data set [60] was downloaded from https://gene.ai.tencent.com/SpatialOmics/dataset?datasetID=27, containing 6 tissue slices. The human cerebellum 10× Visium data set was downloaded from https://www.10xgenomics.com/datasets/human-cerebellum-whole-transcriptome-analysis-1-standard-1-2-0, containing 1 tissue slice. The human breast cancer 10× Visium data set was downloaded from https://www.10xgenomics.com/datasets/, containing 3 tissue slices, named block A section 1, block A section 2, and Fresh Frozen Whole Transcriptome. The human breast 10× Visium data set [61] was downloaded from https://navinlabcode.github.io/HumanBreastCellAtlas.github.io/index.html, containing 10 tissue slices from 4 patients. The human heart 10× Visium data set was downloaded from https://www.10xgenomics.com/datasets/human-heart-1-standard-1-1-0, containing 1 tissue slice. The chicken heart 10× Visium data set [62] was downloaded from https://github.com/madhavmantri/chicken_heart, containing 4 tissue slices. The human cardiac niches 10× Visium data set [63] was downloaded from https://www.heartcellatlas.org, containing 42 tissue slices. The mouse intestine 10× Visium data set [64] was downloaded from https://gene.ai.tencent.com/SpatialOmics/dataset?datasetID=3, containing 4 tissue slices. The human small intestine 10× Visium data set [65] was downloaded from https://data.mendeley.com/datasets/4w6krnywhn/1, containing 14 tissue slices. The human colon 10× Visium data set [65] was downloaded from https://data.mendeley.com/datasets/4w6krnywhn/1, containing 6 tissue slices. The human intestine 10× Visium data set [66] was downloaded from GSE158328, containing 8 tissue slices. The human intestine cancer 10× Visium data set was downloaded from https://www.10xgenomics.com/datasets/human-intestine-cancer-1-standard, containing 1 tissue slice. The adult mouse kidney (FFPE) 10× Visium data set was downloaded from https://www.10xgenomics.com/resources/datasets/adult-mouse-kidney-ffpe-1-standard-1-3-0, containing 1 tissue slice. The mouse kidney (Coronal) 10× Visium data set was downloaded from https://www.10xgenomics.com/datasets/mouse-kidney-section-coronal-1-standard-1-1-0, containing 1 tissue slice. The human kidney 10× Visium data set was downloaded from https://www.10xgenomics.com/datasets/human-kidney-11-mm-capture-area-ffpe-2-standard, containing 1 tissue slice. The human kidney organoid 10× Visium data set [60] was downloaded from https://doi.org/10.17632/xjtv62ncwr.1, containing 3 tissue slices. The mouse liver 10× Visium data set [67] was downloaded from https://livercellatlas.org/, containing 8 tissue slices. The human liver 10× Visium data set [67] was downloaded from https://livercellatlas.org/, containing 5 tissue slices. The human lung 10× Visium data set [68] was downloaded from https://fetal-lung.cellgeni.sanger.ac.uk/, containing 11 tissue slices. The human lung organoid 10× Visium data set [60] was downloaded from https://doi.org/10.17632/xjtv62ncwr.1, containing 4 tissue slices. The human squamous cell carcinoma 10× Visium data set [69] was downloaded from https://doi.org/10.17632/2bh5fchcv6.1, containing 4 tissue slices. The mouse brain ST data set [70] was downloaded from GSE147747, containing 35 tissue slices. The mouse olfactory bulb seqFISH+ data set [19] was downloaded from https://github.com/CaiGroup/seqFISH-PLUS, containing 7 tissue slices. The mouse sub‐ventricular zone seqFISH+ data set [19] was downloaded from https://github.com/CaiGroup/seqFISH-PLUS, containing 7 tissue slices. The mouse medial prefrontal cortex STARmap data set [21] was downloaded from https://github.com/zhengli09/BASS-Analysis/blob/master/data/starmap_mpfc.RData, containing 3 tissue slices. The mouse neocortex V1 STARmap data set [21] was downloaded from https://zenodo.org/record/7830764#.ZDpObi-1HUI, containing 2 tissue slices. The mouse primary motor cortex MERFISH data set [71] was downloaded from https://caltech.box.com/shared/static/dzqt6ryytmjbgyai356s1z0phtnsbaol.gz. A total of 11 tissue slices from sample 1 and sample 2 were utilized. The mouse hypothalamus MERFISH data set [72] was downloaded from https://datadryad.org/stash/dataset/doi:10.5061/dryad.8t8s248. A total of 30 tissue slices from the naive female animals (*Animal_ID*: 1, 2, and 3) were utilized. The mouse brain Slide‐seq data set [16] was downloaded from https://gene.ai.tencent.com/SpatialOmics/dataset?datasetID=1, containing 5 tissue slices. The mouse olfactory bulb Slide‐seq data set [73] was downloaded from https://db.cngb.org/stomics/datasets/STDS0000172, containing 20 tissue slices from sample 1. The human lung Slide‐seq data set [74] was downloaded from https://cellxgene.cziscience.com/collections/02b01703-bf1b-48de-b99a-23bef8cccc81, containing 7 tissue slices. The human lymph node Slide‐seq data set [74] was downloaded from https://cellxgene.cziscience.com/collections/02b01703-bf1b-48de-b99a-23bef8cccc81, containing 7 tissue slices. The human tonsil Slide‐seq data set [74] was downloaded from https://cellxgene.cziscience.com/collections/02b01703-bf1b-48de-b99a-23bef8cccc81, containing 1 tissue slice. The mouse spleen Slide‐seq data set [74] was downloaded from https://cellxgene.cziscience.com/collections/02b01703-bf1b-48de-b99a-23bef8cccc81, containing 1 tissue slice. The mouse testis Slide‐seq data set [75] was downloaded from https://www.dropbox.com/scl/fi/dr10k6x92hxdnuijgdobn/Testis_Slide-seq_Data.zip?rlkey=yy5odlkqqynfped6rixwcnk5l&e=1&dl=0, containing 6 tissue slices. The human melanoma brain metastasis Slide‐seq data set [76] was downloaded from https://gene.ai.tencent.com/SpatialOmics/dataset?datasetID=119, containing 15 tissue slices. The human kidney Slide‐seq data set [77] was downloaded from https://cellxgene.cziscience.com/collections/8e880741-bf9a-4c8e-9227-934204631d2a, containing 10 tissue slices. The mouse kidney Slide‐seq data set [77] was downloaded from https://cellxgene.cziscience.com/collections/8e880741-bf9a-4c8e-9227-934204631d2a, containing 30 tissue slices. The mouse olfactory bulb Stereo‐seq data set [17] was downloaded from https://db.cngb.org/stomics/mosta/download/, containing 2 tissue slices. The adult mouse hemi‐brain Stereo‐seq data set [17] was downloaded from https://db.cngb.org/stomics/mosta/download/, containing 3 tissue slices. The mouse brain 10× Visium HD data set was downloaded from https://www.10xgenomics.com/, containing 3 tissue slices. The human colorectal cancer 10× Visium HD data set [18] was downloaded from https://www.10xgenomics.com/platforms/visium/product-family/dataset-human-crc, containing 3 tissue slices. The mouse brain Xenium data set [57] was downloaded from https://www.10xgenomics.com/datasets/fresh-frozen-mouse-brain-replicates-1-standard, containing 1 tissue slice. The human breast cancer Xenium data set [23] was downloaded from https://www.10xgenomics.com/products/xenium-in-situ/preview-dataset-human-breast, containing 2 tissue slices. The mouse brain CosMx data set [22] was downloaded from https://nanostring.com/products/cosmx-spatial-molecular-imager/ffpe-dataset/cosmx-smi-mouse-brain-ffpe-dataset/, containing 2 tissue slices. The non‐small‐cell lung cancer CosMx data set [78] was downloaded from https://zenodo.org/records/15240431, containing 4 tissue slices.

### Data preprocessing

For each data set, the gene expression matrix and spatial coordinates were required as inputs. H&E images, available for 10× Visium and ST platforms, were optionally used by DeepST, SpaGCN, and stLearn. Spots annotated as “unknown” in the original ground truth, as well as genes expressed at 0 across all spots, were removed. Due to computing memory limitations, most methods returned “out of memory” errors on large‐scale datasets. Therefore, for data from Stereo‐seq, Visium HD, CosMx, and Xenium, original slices were partitioned into smaller subsets, which were then treated as independent objects for evaluation.

Each method optionally carried out normalization, logarithmic conversion, highly expressed gene selection, dimensionality reduction, and related preprocessing steps. The specific parameter settings are described in the supplementary information. For clustering, we set the expected number of clusters equal to the number of clusters in the ground truth. For methods that instead require a resolution parameter (e.g., DeepST, SpaceFlow), we tuned the resolution until the number of clusters matched the expected number.

To systematically evaluate the effect of preprocessing steps, we compiled a set of strategies and tested 200 different parameter combinations on DLPFC 10× Visium datasets, covering five key components: normalization (applied or not), log transformation (applied or not), gene selection strategy (using all genes, HVGs, or SVGs), standardization (applied or not), and dimension reduction (not applied, 15PCs, 20PCs, 50PCs, and 200 PCs). To assess the contribution of individual preprocessing steps to clustering accuracy, we applied ANOVA analysis of variance within a linear modeling framework, treating ARI as the response variable and each preprocessing step as a categorical predictor. We further applied the optimized pipeline to Human Small Intestine datasets to demonstrate its performance.

### Simulation datasets of variable replicates

We used scCube to simulate multiple replicates based on the DLPFC 10× Visium and hypothalamus MERFISH datasets. Specifically, the DLPFC data set included three donors, with four slices obtained per donor that shared the same spatial domains. For each donor, the model was trained on the gene expression matrix from one slice and projected onto another slice from the same donor, generating a total of 48 simulated datasets. Using the same strategy, we generated 78 simulated datasets from the hypothalamus MERFISH data set.

To evaluate the effect of altered spatial domain adjacency, we further applied scCube to the DLPFC slice of *151673*. Here, new spatial configurations were created by randomly removing one of the original seven spatial domains and filling the vacant location with spots from adjacent spatial domains, producing 12 simulated datasets from the pre‐trained model. Additionally, we simulated the addition of missing spatial domains. For example, in DLPFC slice *151670*, where Layer 2 was absent, we incorporated expression profiles of Layer 2 sampled from other tissue sections and placed them adjacent to Layer 3, generating 16 simulated slices.

### Simulation datasets of variable spatial patterns

SRTsim is a simulator specifically designed for SRT data, allowing for interactive customization of cluster distribution patterns and generating gene expression profiles for each spot accordingly.

Simulation 1 (spot number): We simulated datasets with varying numbers of spots. Spots were arranged in a regular grid resembling ST technology, with total counts of 1024, 2025, 3025, 4096, 5041, 5929, 7056, 7921, 9025, and 10,000. These spots were divided into four linear regions to mimic cortical structure. Each spot was assigned a gene expression matrix of 5,000 genes. Default parameters were used (Zero% = 0.05, Dispersion = 0.5, Mean = 2).

Simulation 2 (gene number): We simulated datasets with varying numbers of genes. A regular grid of 3000 spots was divided into four linear clusters. The number of genes was set to 500, 1000, 2000, 3000, 4000, 5000, 6000, 7000, 8000, and 9000. Default parameters were used (Zero% = 0.05, Dispersion = 0.5, Mean = 2).

Simulation 3 (sparsity): We simulated datasets with different levels of sparsity. A regular grid of 3000 spots and 5,000 genes was divided into four linear clusters. Sparsity was varied from 0.291 to 0.925 in 10 increments (0.291, 0.328, 0.403, 0.477, 0.552, 0.627, 0.701, 0.776, 0.851, and 0.925). Other parameters were set at default (Dispersion = 0.5, Mean = 2).

Simulation 4 (cluster number): We simulated datasets with varying numbers of clusters. A grid of 3000 spots and 5000 genes was divided into 2, 4, 6, 8, 10, 12, 14, and 16 linear clusters. The other parameters were set at default (Zero% = 0.05, Dispersion = 0.5, Mean = 2).

Simulation 5 (cluster shape): We simulated datasets with personalized cluster shapes. A gene expression matrix with 5000 genes was generated for randomly distributed spots assigned to four irregular clusters. Default parameters were used (Zero% = 0.05, Dispersion = 0.5, Mean = 2). To enable compatibility with BayesSpace, which requires regularly arranged spatial coordinates, spatial coordinates were scaled and mapped to the nearest position on a 100 × 100 grid, allowing at most one spot per grid cell. This preserved expression profiles while imposing a sparse spatial structure.

Simulation 6 (complex spatial patterns): Using scCube, we simulated datasets under parametric control with a built‐in data set containing 2000 genes across 408 cells. The parameter δ (connectivity) was set to 5, 15, 25, 35, 45, and λ (fuzziness) to 0.2, 0.4, 0.6, 0.8, 1.0, generating diverse random spatial patterns. Parameters were set as platform = “Visium” and n_cell = 0.8 in the function *model. generate_spot_data_random*. Other parameters were set at default.

### Benchmark evaluation metrics

The ARI is the extension of the Rand Index, correcting for random consistency, which is designed to quantify pairwise similarity between two clustering results, typically between the ground truth and predicted clustering. ARI value ranges from −1 to 1, where a value closer to 1 indicates more similar clustering results, and 0 represents the expected agreement by chance. ARI is calculated as Equation (1):

(1) ARI=∑ijnij2−∑iai2∑jbj2/n212∑iai2+∑jbj2−∑iai2∑jbj2/n2,

where $n_{\mathrm{ij}}$ is the number of spots in both the true cluster i and the predicted cluster j, $a_{i}$ is the number of spots in the true cluster i, $b_{j}$ is the number of spots in the predicted cluster j, and n is the total number of spots.

NMI measures the dependency between the ground truth and predicted clustering based on Mutual Information, normalized to avoid biases caused by differences in the sizes of the cluster sets. NMI value ranges from 0 to 1, where 1 indicates perfect agreement and 0 indicates random clustering. NMI is computed as Equations ((2), (3), (4)):

(2) $$\mathrm{MI}\left(A,\;B\right)=\sum_{i}^{\left|A\right|}\sum_{j}^{\left|B\right|}P\left(A_{i},B_{j}\right)\log\left(\frac{P\left(A_{i},B_{j}\right)}{P\left(A_{i}\right)P\left(B_{j}\right)}\right),$$

(3) $$H\left(A\right)=-\sum_{i}^{\left|A\right|}P\left(A_{i}\right)\log P\left(A_{i}\right),$$

(4) $$\mathrm{NMI}\left(A,B\right)=\frac{\mathrm{MI}\left(A,\;B\right)}{\sqrt{H\left(A\right)H\left(B\right)}},$$

where $P\left(A_{i}\right)$ and $P\left(B_{j}\right)$ represent the probabilities of each cluster in ground truth (*A*) and predicted result (*B*), and $P\left(A_{i},B_{j}\right)$ represents the joint probability of cluster $A_{i}$ and $B_{j}$ occurring together, H(A) and H(B) are the entropies of ground truth and predicted clustering.

FMI is an external evaluation score that is used to determine the similarity between two clustering, emphasizing the precision and recall of clustered pairs. FMI value ranges from 0 to 1, where a higher value indicates a greater similarity between the ground truth and predicted clustering. FMI is calculated as Equation (5):

(5) $$\mathrm{FMI}=\sqrt{\mathrm{PPV}\times \mathrm{TPR}}=\sqrt{\frac{\mathrm{TP}}{\mathrm{TP}+\mathrm{FP}}\times \frac{\mathrm{TP}}{\mathrm{TP}+\mathrm{FN}}},$$

where TPR is the true positive rate, also called sensitivity or recall, and PPV is the positive predictive rate, also known as precision. *TP* is the number of pairs that are in the same cluster in both the ground truth and predicted clustering, *TN* is the number of pairs that are in different clusters in both the ground truth and predicted clustering, *FN* is the number of pairs that are in the same cluster in the ground truth but in different clusters in the predicted clustering, and *FP* is the number of pairs that are in different clusters in the ground truth but in the same cluster in the predicted clustering.

Purity is an asymmetric clustering evaluation index that assesses how well the clustering results match the ground truth by counting the proportion of the predominant ground truth among the spots in each cluster. Purity value ranges from 0 to 1, where 1 represents a perfect cluster and 0 represents a completely unrelated cluster. Purity is computed as Equation (6):

(6) $$\mathrm{Purity}=\frac{1}{n}\sum_{j}\max_{i}|A_{i}\cap B_{j}|,$$

where $A_{i}$ is the cluster i in ground truth and $B_{j}$ is the predicted cluster j, n is the total number of spots.

Homogeneity evaluates the internal consistency of the predicted clusters, ranging from 0 to 1, and the closer the value is to 1, the better the clustering results, indicating that the spots within a predicted cluster share the same ground truth. Mathematically, Homogeneity is defined as Equations (7) and (8):

(7) $$\mathrm{Homogeneity}=1-\frac{H\left(A|B\right)}{H\left(A\right)},$$

(8) $$H\left(A|B\right)=-\sum_{j}^{\left|B\right|}P\left(B_{j}\right)\sum_{i}^{\left|A\right|}P\left(A_{i}|B_{j}\right)\log P\left(A_{i}|B_{j}\right),$$

where H(A) is the entropy of ground truth, H(A|B) represents the conditional entropy of the ground truth A given the predicted clustering B, measures the remaining uncertainty in the ground truth after knowing the predicted clustering.

Completeness evaluates the cohesion of the ground truth, which requires that all spots belonging to the same ground truth are assigned to the same cluster, preventing ground truth fragmentation. Mathematically, Completeness is defined as Equations (9) and (10):

(9) $$\mathrm{Completeness}=1-\frac{H\left(B|A\right)}{H\left(B\right)},$$

(10) $$H\left(B|A\right)=-\sum_{i}^{\left|A\right|}P\left(A_{i}\right)\sum_{j}^{\left|B\right|}P\left(B_{j}|A_{i}\right)\log P\left(B_{j}|A_{i}\right),$$

where H(B) is the entropy of predicted clustering. H(B|A) represents the conditional entropy of the predicted clustering B given the ground truth A, measuring how much uncertainty remains about the predicted clustering after knowing the ground truth.

In this study, we evaluated the accuracy of spatial clustering methods using multiple quantitative metrics as described above. To provide an overall accuracy score, we ranked the clustering methods in descending order for each metric and calculated the average rank across all evaluation metrics, such that methods with lower average ranks (indicating better performance) received lower accuracy scores. This approach ensures that each metric contributes equally to the overall accuracy score, while also providing a comprehensive evaluation of clustering accuracy across different aspects, as with previous benchmark studies [79].

(11) $$\mathrm{Accuracy}\;\mathrm{Score}=\frac{1}{6}\left({\mathrm{Rank}}_{\mathrm{ARI}}+{\mathrm{Rank}}_{\mathrm{NMI}}+{\mathrm{Rank}}_{\mathrm{FMI}}\;+{\mathrm{Rank}}_{\mathrm{Purity}}+{\mathrm{Rank}}_{\mathrm{Hom}}+{\mathrm{Rank}}_{\mathrm{Com}}\right).$$

CHAOS score is adopted to quantify image segmentation performance in mass spectrometry imaging [80, 81], which measures the spatial continuity of the detected spatial clusters. To calculate the CHAOS score, we first create a one‐nearest‐neighbor (1NN) graph for the spots in each spatial cluster by connecting each spot with its nearest neighbor and calculating the edge weight between spots as in Equation (12):

(12) $$\begin{array}{c} w_{\mathrm{kij}}=\left\{\begin{array}{l} d_{kij},\mathrm{if}\;\mathrm{spot}\;i\;\mathrm{and}\;j\;\mathrm{are}\;\mathrm{connected}\;\mathrm{in}\;\mathrm{the}\;1\mathrm{NN}\;\mathrm{graph}\;\mathrm{in}\;\mathrm{cluster}\;k\\ 0,\;\mathrm{otherwise} \end{array}\right., \end{array}$$

where $d_{\mathrm{kij}}$ is the Euclidean distance between the spot i and j in cluster k. Then, the CHAOS score is computed as the mean distance of the graph edges in the 1NN graph as Equation (13):

(13) $$\mathrm{CHAOS}=\frac{1}{N}\sum_{k=1}^{K}\sum_{i,j}^{n_{k}}w_{\mathrm{kij}},$$

where N is the total number of spots. K is the total number of spatial clusters. $n_{k}$ is the number of spots in the *k*‐th spatial cluster. A lower CHAOS score indicates better spatial continuity.

The PAS score measures the spatial dispersion of spots that share the same cluster label. It is calculated as the proportion of spots with a cluster label that is different from at least six of its neighboring ten spots. The PAS score ranges from 0 to 1, where a small PAS score indicates a better continuity of detected spatial clusters.

The ASW score measures how similar a spot is to its predicted cluster compared to other clusters. The value of the ASW score ranges from −1 to 1, with higher values indicating better spatial coherence. We define silhouette width (SW) first for each spot and compute ASW by averaging SW across all spots. SW is computed as Equation (2):

(14) $$\mathrm{SW}=\frac{b-a}{\max\left(a,b\right)},$$

where a is the mean distance between a spot and all other spots within the same spatial cluster. b is the mean distance between a spot and all other spots within the nearest cluster.

For Python‐based methods, we used the built‐in *time* and *tracemalloc* packages. The runtime was recorded by measuring the wall‐clock time before and after the execution of the clustering algorithm using *time.time()*. Peak memory usage during execution was obtained using *tracemalloc.get_traced_memory()*. For R‐based methods, we used *Sys.time()* to capture runtime and *gc()* to estimate memory usage.

### Statistics

Each evaluation was performed in five replicated runs per sample in parallel, and the average value was calculated for subsequent analyses. For each group sample, we calculated the median, interquartile range, and 95% confidence intervals. All evaluation metrics were computed using the *sklearn* package, and Pearson correlation coefficients were calculated with the *scipy* package. Python (version 3.8.5) and R (version 4.3.0) are used for the statistical analysis and figure generation.

## AUTHOR CONTRIBUTIONS

**Renjie Chen**: Writing—original draft; software; data curation; validation. **Yue Yao**: Visualization; methodology. **Jingyang Qian**: Methodology; visualization. **Xin Peng**: Writing—review and editing; funding acquisition; conceptualization; project administration. **Xin Shao**: Writing—review and editing; funding acquisition; conceptualization; project administration. **Xiaohui Fan**: Writing—review and editing; funding acquisition; conceptualization; project administration.

## CONFLICT OF INTEREST STATEMENT

The authors declare no conflicts of interest.

## ETHICS STATEMENT

No animals or humans were involved in this study.

## Supporting information


**Figure S1:** Evaluation of ground truth reliability.
**Figure S2:** Evaluation on the 10× Visium dataset slice *Mouse_Brain_Section_Coronal*.
**Figure S3:** Evaluation on the ST dataset slice *Mouse_Brain_20A*.
**Figure S4:** Evaluation on the seqFISH+ dataset slice *Mouse Olfactory Bulb View0*.
**Figure S5:** Evaluation on the STARmap dataset slice *Mouse Visual Cortex 20180410_BY3_1kgenes*.
**Figure S6:** Evaluation on the MERFISH dataset slice *Hypothalamus Animal1 Bregma‐0.04*.
**Figure S7:** Evaluation on the CosMx dataset slice *Mouse Brain Hemisphere (sub4)*.
**Figure S8:** The other quantitative accuracy metrics of methods on variable technologies.
**Figure S9:** The overall accuracy score of the methods on variable technologies.
**Figure S10:** The stability of spatial clustering methods across all datasets.
**Figure S11:** Computational resource requirements of spatial clustering methods across technologies.
**Figure S12:** Performance comparison of methods on cell type clustering and spatial niche detection.
**Figure S13:** Performance variation of clustering methods across organs.
**Figure S14:** Spatial continuity varies across organs in 10× Visium datasets.
**Figure S15:** The other accuracy metrics of the methods on the variable organs.
**Figure S16:** Performance comparison with SRT datasets from kidney and skin organs.
**Figure S17:** The overall performance of the methods on variable organs.
**Figure S18:** The quantitative evaluation metrics of spatial continuity on variable organs.
**Figure S19:** Computational resource requirements of spatial clustering methods across organs.
**Figure S20:** Spatial continuity varies across organs in Slide‐seq datasets.
**Figure S21:** Performance comparison of methods on real datasets and simulated datasets.
**Figure S22:** Computational resource requirements of spatial clustering methods across simulated datasets from variable spatial patterns.
**Figure S23:** Contribution of data characteristics to clustering accuracy (ARI).
**Figure S24:** Pearson correlation of parameters, lambda and delta, to ARI.
**Figure S25:** Top 8 preprocessing pipeline of methods (Banksy, BASS, BayesSpace, CCST, CellCharter, DeepST, and GraphST).
**Figure S26:** Top 8 preprocessing pipeline of methods (PRECAST, SEDR, SpaceFlow, SpaGCN, SpatialMGCN, STAGATE, and stLearn).
**Figure S27:** Evaluation of preprocessing steps and parameters on clustering accuracy.
**Figure S28:** Evaluation of optimal preprocessing pipelines on the DLPFC dataset and the Human Small Intestine dataset.
**Figure S29:** Comparison of multi‐slice integration and single‐sample analysis on clustering accuracy.
**Figure S30:** Overall accuracy of clustering methods across datasets.
**Figure S31:** Overall accuracy of clustering methods in diverse contexts.

**Table S1:** Datasets used in benchmark.
**Table S2:** Recommended clustering methods under different scenarios, summarized by technology, organ, and biological replicates.

## Data Availability

The data that support the findings of this study are available in the supplementary material of this article. The datasets used in this study are publicly accessible as described above, as well as in Table S1. The data and scripts used are saved in GitHub (https://github.com/ZJUFanLab/SRTBenchmark). Supplementary materials (figures, methods, tables, graphical abstract, slides, videos, Chinese translated version, and update materials) may be found in the online DOI or iMeta Science http://www.imeta.science/.
