# Supplementary material for: A comprehensive benchmarking for spatially resolved transcriptomics clustering methods across variable technologies, organs, and replicates
Source: Imeta. 2025 Oct 9;4(6):e70084. doi: 10.1002/imt2.70084 (PMC12747554; doi:10.1002/imt2.70084)
Supplement: Supplementary file 1 — Figure S1: Evaluation of ground truth reliability. Figure S2: Evaluation on the 10× Visium dataset slice Mouse_Brain_Section_Coronal. Figure S3: Evaluation on the ST dataset slice Mouse_Brain_20A. Figure S4: Evaluation on the seqFISH+ dataset slice Mouse Olfactory Bulb View0. Figure S5: Evaluation on the STARmap dataset slice Mouse Visual Cortex 20180410_BY3_1kgenes. Figure S6: Evaluation on the MERFISH dataset slice Hypothalamus Animal1 Bregma‐0.04. Figure S7: Evaluation on the CosMx dataset slice Mouse Brain Hemisphere (sub4). Figure S8: The other quantitative accuracy metrics of methods on variable technologies. Figure S9: The overall accuracy score of the methods on variable technologies. Figure S10: The stability of spatial clustering methods across all datasets. Figure S11: Computational resource requirements of spatial clustering methods across technologies. Figure S12: Performance comparison of methods on cell type clustering and spatial niche detection. Figure S13: Performance variation of clustering methods across organs. Figure S14: Spatial continuity varies across organs in 10× Visium datasets. Figure S15: The other accuracy metrics of the methods on the variable organs. Figure S16: Performance comparison with SRT datasets from kidney and skin organs. Figure S17: The overall performance of the methods on variable organs. Figure S18: The quantitative evaluation metrics of spatial continuity on variable organs. Figure S19: Computational resource requirements of spatial clustering methods across organs. Figure S20: Spatial continuity varies across organs in Slide‐seq datasets. Figure S21: Performance comparison of methods on real datasets and simulated datasets. Figure S22: Computational resource requirements of spatial clustering methods across simulated datasets from variable spatial patterns. Figure S23: Contribution of data characteristics to clustering accuracy (ARI). Figure S24: Pearson correlation of parameters, lambda and delta, to ARI. Figure S25: [file IMT2-4-e70084-s002.docx]

**Supporting information to**

**A comprehensive benchmarking for spatially resolved transcriptomics clustering methods across variable technologies, organs, and replicates**

**Running title**: Benchmarking spatially resolved transcriptomics clustering methods

Renjie Chen^1,2^, Yue Yao^1,2^, Jingyang Qian^1,2^, Xin Peng^3*^, Xin Shao^1,2,3*^, Xiaohui Fan^1,2,3,4*^

^1^Pharmaceutical Informatics Institute, College of Pharmaceutical Sciences, Zhejiang University, Hangzhou 310058, China

^2^State Key Laboratory of Chinese Medicine Modernization, Innovation Center of Yangtze River Delta, Zhejiang University, Jiaxing 314103, China

^3^Ningbo Municipal Hospital of TCM, Affiliated Hospital of Zhejiang Chinese Medical University, Ningbo 315000, China

^4^Zhejiang Key Laboratory of Precision Diagnosis and Therapy for Major Gynecological Diseases, Women's Hospital, Zhejiang University School of Medicine, Hangzhou 310006, China

*Correspondence: pengx@nit.zju.edu.cn (Xin Peng), xin_shao@zju.edu.cn (Xin Shao), fanxh@zju.edu.cn (Xiaohui Fan).

**Supplementary methods**

**Performance comparison of spatial clustering methods**

For BASS [1], we normalized the data with a log2 transformation and performed dimension reduction with PCA in 3000 top-expressed genes in 10× Visium and ST datasets or all genes in seqFISH+, MERFISH, and STARmap datasets, 20 principal components chosen for training the BASS model. Banksy [2] normalized gene expression and selected the top 2000 highly variable genes. The physical-space neighborhood graph was constructed with a *k*-nearest neighbor approach, where k was set to 18 in 10× Visium and 15 in others. To compute the neighbor-augmented matrix, we used a setting of *λ* = 0.2 for cell typing and *λ* = 0.8 for domain segmentation. Next, we used PCA to reduce the dimensionality and retained the top 20 principal components. For BayesSpace [3], we set the parameters “n.PCs = 15” and “n.HVGs = 2000” in preprocessing. The spatial prior parameter “gamma” was set to 2 for ST data and to 3 for 10× Visium data. For CCST [4], we set the output dimension of PCA to 200 in the function *adata_preprocess*. The value of the hyperparameter “lambda_I” for weighing intracellular and extracellular information was set to 0.3 in ST and 10× Visium datasets and 0.8 in MERFISH. In the Deep Graph Infomax model, we trained for 5000 epochs and set the number of hidden channels to 256 as the default. For CellCharter [5], we selected the top 5000 highly variable genes with normalized and log-transformed data, then used scVI to perform dimensionality reduction and batch effect removal. For DeepST [6], we used morphological images as input for ST and 10× Visium datasets. In the function *deepen._get_augment*, “LinearRegress” mode applied to 10× Visium, and the remaining datasets selected “BallTree” as recommended. The parameter “pca_n_comps” was set to 200 for dimension reduction and reduced accordingly when the number of spots or genes was less than 200. For GraphST [7], we used the “mclust” algorithm to cluster the generated embeddings and an optional refinement step for the DLPFC dataset. For PRECAST [8], in the function *AddAdjList*, we selected the built-in arguments “platform” to “Visium”, “ST”, and “Other_SRT” according to SRT technologies to add the adjacency matrix list. For SEDR [9], we selected 2000 highly variable genes and used the PCA function from the *sklearn* package. In the function *graph_construction*, the parameter “n” was set to 12 to define the number of adjacent spots for 10× Visium datasets and set to 6 for Stereo-seq datasets. For SpaceFlow [10], we created a SpaceFlow object and selected the top 3000 highly variable genes in preprocessing. After training a spatially regularized deep graph network model to learn a low-dimensional embedding with default parameters, we identified the spatial clusters based on the Leiden algorithm, where the resolution parameter was searched to match the number of ground truth. For SpaGCN [11], we integrated gene expression and histology into a graph and performed an optional refinement step for 10× Visium and ST datasets, where the parameter “shape” was set to “hexagon” for 10× Visium datasets and “square” for ST datasets. The parameter “max_run” was expanded to search appropriate resolution for the initial Louvain clustering in the function *spg.search_res*. For SpatialMGCN [12], we set the parameters “alpha”, “beta”, and “gamma” to 1, 10, and 0.1 respectively, in the training model for 10× Visium datasets. The model takes the 3000 highly variable genes as input, inputs all genes, and modifies the parameter “nfeat” accordingly for seqFISH+, STARmap, and MERFISH datasets. For STAGATE [13], the parameter “rad_cutoff” was searched based on the neighbors per cell to calculate the spatial graph. Then we used the cell type-aware module for 10× Visium and ST datasets, setting the parameters “alpha = 0.5”, “pre_resolution = 0.2”, “n_epochs = 1000” in the function *STAGATE.train_STAGATE*, and “alpha = 0” for other datasets. For stLearn [14], the parameter “weights” was set to “weights_matrix_pd_md” for normalization, combining spatial distance and morphological distance for ST and 10× Visium datasets. In other datasets, the first 50 principal components were selected for K-means clustering.

**REFERENCES**

1. Li, Zheng, Xiang Zhou. 2022. “BASS: multi-scale and multi-sample analysis enables accurate cell type clustering and spatial domain detection in spatial transcriptomic studies.” *Genome Biology* 23: 168. https://doi.org/10.1186/s13059-022-02734-7

2. Singhal, Vipul, Nigel Chou, Joseph Lee, Yifei Yue, Jinyue Liu, Wan Kee Chock, Li Lin, et al. 2024. “BANKSY unifies cell typing and tissue domain segmentation for scalable spatial omics data analysis.” *Nature Genetics* 56: 431441. https://doi.org/10.1038/s41588-024-01664-3

3. Zhao, Edward, Matthew R. Stone, Xing Ren, Jamie Guenthoer, Kimberly S. Smythe, Thomas Pulliam, Stephen R. Williams, et al. 2021. “Spatial transcriptomics at subspot resolution with BayesSpace.” *Nature Biotechnology* 39: 1375−1384. https://doi.org/10.1038/s41587-021-00935-2

4. Li, Jiachen, Siheng Chen, Xiaoyong Pan, Ye Yuan, Hong-Bin Shen. 2022. “Cell clustering for spatial transcriptomics data with graph neural networks.” *Nature Computational Science* 2: 399−408. https://doi.org/10.1038/s43588-022-00266-5

5. Varrone, Marco, Daniele Tavernari, Albert Santamaria-Martínez, Logan A. Walsh, Giovanni Ciriello. 2024. “CellCharter reveals spatial cell niches associated with tissue remodeling and cell plasticity.” *Nature Genetics* 56: 74−84. https://doi.org/10.1038/s41588-023-01588-4

6. Xu, Chang, Xiyun Jin, Songren Wei, Pingping Wang, Meng Luo, Zhaochun Xu, Wenyi Yang, et al. 2022. “DeepST: identifying spatial domains in spatial transcriptomics by deep learning.” *Nucleic Acids Research* 50: e131−e131. https://doi.org/10.1093/nar/gkac901

7. Long, Yahui, Kok Siong Ang, Mengwei Li, Kian Long Kelvin Chong, Raman Sethi, Chengwei Zhong, Hang Xu, et al. 2023. “Spatially informed clustering, integration, and deconvolution of spatial transcriptomics with GraphST.” *Nature Communications* 14: 1155. https://doi.org/10.1038/s41467-023-36796-3

8. Liu, Wei, Xu Liao, Ziye Luo, Yi Yang, Mai Chan Lau, Yuling Jiao, Xingjie Shi, et al. 2023. “Probabilistic embedding, clustering, and alignment for integrating spatial transcriptomics data with PRECAST.” *Nature Communications* 14: 296. https://doi.org/10.1038/s41467-023-35947-w

9. Xu, Hang, Huazhu Fu, Yahui Long, Kok Siong Ang, Raman Sethi, Kelvin Chong, Mengwei Li, et al. 2024. “Unsupervised spatially embedded deep representation of spatial transcriptomics.” *Genome Medicine* 16: 12. https://doi.org/10.1186/s13073-024-01283-x

10. Ren, Honglei, Benjamin L. Walker, Zixuan Cang, Qing Nie. 2022. “Identifying multicellular spatiotemporal organization of cells with SpaceFlow.” *Nature Communications* 13: 4076. https://doi.org/10.1038/s41467-022-31739-w

11. Hu, Jian, Xiangjie Li, Kyle Coleman, Amelia Schroeder, Nan Ma, David J. Irwin, Edward B. Lee, Russell T. Shinohara, Mingyao Li. 2021. “SpaGCN: Integrating gene expression, spatial location and histology to identify spatial domains and spatially variable genes by graph convolutional network.” *Nature Methods* 18: 1342−1351. https://doi.org/10.1038/s41592-021-01255-8

12. Wang, Bo, Jiawei Luo, Ying Liu, Wanwan Shi, Zehao Xiong, Cong Shen, Yahui Long. 2023. “Spatial-MGCN: a novel multi-view graph convolutional network for identifying spatial domains with attention mechanism.” *Briefings in Bioinformatics* 24: bbad262. https://doi.org/10.1093/bib/bbad262

13. Dong, Kangning, Shihua Zhang. 2022. “Deciphering spatial domains from spatially resolved transcriptomics with an adaptive graph attention auto-encoder.” *Nature Communications* 13: 1739. https://doi.org/10.1038/s41467-022-29439-6

14. Pham, Duy, Xiao Tan, Brad Balderson, Jun Xu, Laura F. Grice, Sohye Yoon, Emily F. Willis, et al. 2023. “Robust mapping of spatiotemporal trajectories and cell–cell interactions in healthy and diseased tissues.” *Nature Communications* 14: 7739. https://doi.org/10.1038/s41467-023-43120-6

**Supplementary figures**

**
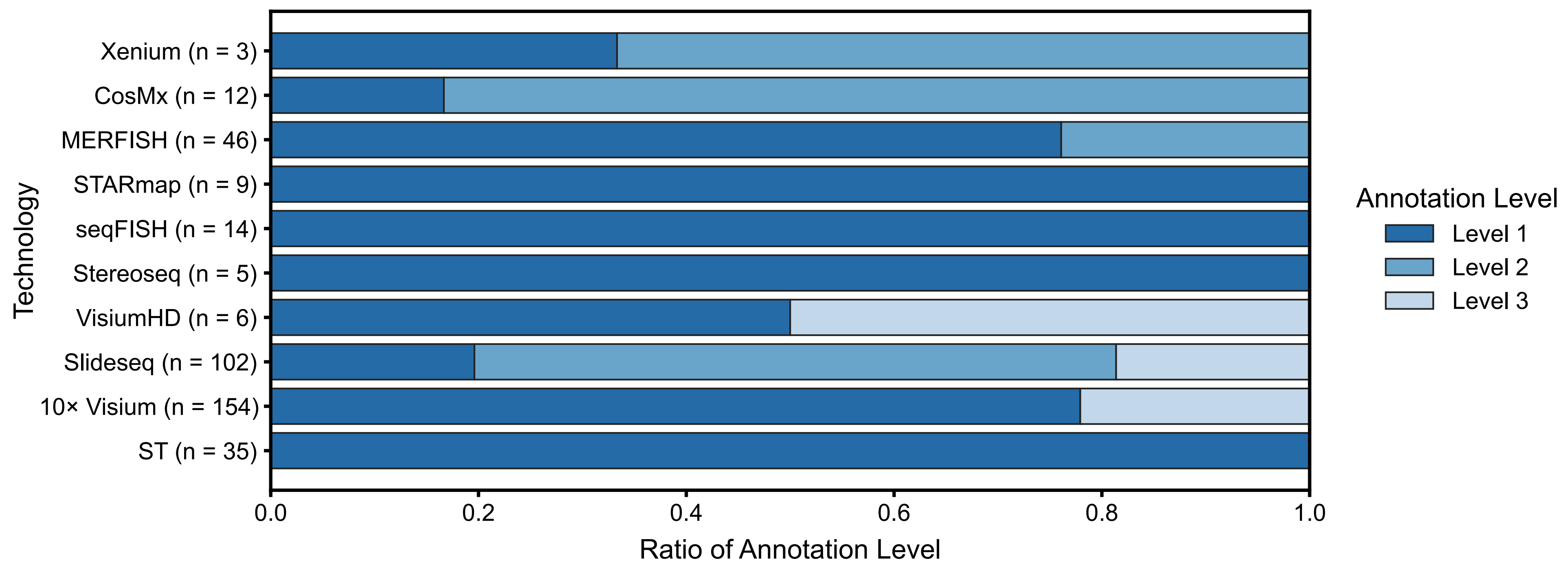
**

**Figure S1** **Evaluation of ground truth reliability.** Proportion of slices with different ground truth confidence levels across all evaluated technologies. The x-axis represents the ratio, and the y-axis represents the methods. Different colors represent different methods. Level 1: Manually annotated based on histological morphology or validated marker genes. These annotations were supported by experimental validation in the original publications and are considered highly reliable. Level 2: Annotations transferred from matched single-cell RNA-seq references using computational tools such as RCTD. These are informative, but they depend on the quality of the reference dataset. Level 3: Annotations generated by unsupervised clustering pipelines, such as graph-based clustering results provided directly from the 10× Genomics websites. These annotations may reflect methodological biases and are considered less reliable.

**Figure S2** **Evaluation on the 10× Visium dataset slice *Mouse_Brain_Section_Coronal*.** This slice is annotated to 15 domains in the ground truth. The ground truth and results of 14 spatial clustering methods are shown. The scores of ARI and NMI are indicated to quantitatively evaluate the accuracy of methods.

**Figure S3** **Evaluation on the ST dataset slice *Mouse_Brain_20A*.** This slice is annotated to 11 domains in the ground truth. The ground truth and results of 14 spatial clustering methods are shown. The scores of ARI and NMI are indicated to quantitatively evaluate the accuracy of methods.

**Figure S4** **Evaluation on the seqFISH+ dataset slice *Mouse Olfactory Bulb View 0*.** This slice is annotated to 9 cell types in the ground truth. The ground truth and results of 13 spatial clustering methods are shown. The scores of ARI and NMI are indicated to quantitatively evaluate the accuracy of methods.

**Figure S5** **Evaluation on the STARmap dataset slice *Mouse Visual Cortex* *20180410_BY3_1kgenes*.** This slice is annotated to 7 domains (A) and 15 cell types (B) in the ground truth. The ground truth and results of 13 spatial clustering methods are shown. The scores of ARI and NMI are indicated to quantitatively evaluate the accuracy of methods.

**Figure S6** **Evaluation on the MERFISH dataset slice *Hypothalamus Animal1 Bregma-0.04*.** This slice is annotated to 8 domains (A) and 9 cell types (B) in the ground truth. The ground truth and results of 13 spatial clustering methods are shown. The scores of ARI and NMI are indicated to quantitatively evaluate the accuracy of methods.

**Figure S7** **Evaluation on the CosMx dataset slice *Mouse Brain* *Hemisphere (sub4)*.** This slice is annotated to 16 spatial niches in the ground truth. The ground truth and results of 13 spatial clustering methods are shown. The scores of ARI and NMI are indicated to quantitatively evaluate the accuracy of methods.

**Figure S8** **The other quantitative accuracy metrics of methods on variable technologies.** The boxplots of comparing methods on all brain datasets of variable technologies with FMI, Purity, Homogeneity, and Completeness. Center line: median; box limits: upper and lower quartiles; whiskers: 1.5× interquartile range (IQR). Color: technologies.

**
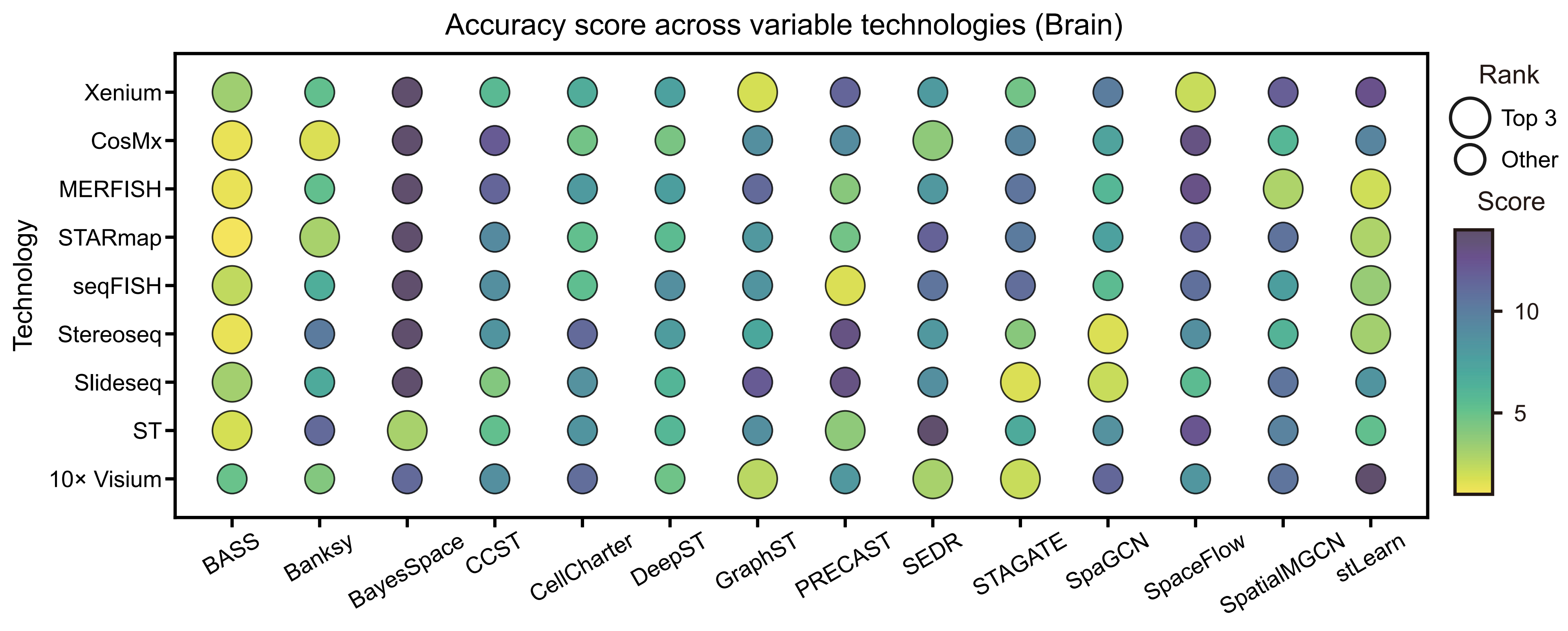
**

**Figure S9** **The overall accuracy score of the methods on variable technologies.** The bubble plot of accuracy scores for 14 methods on all brain datasets of variable technologies, where a lower accuracy score indicates a higher ranking in terms of method accuracy, and larger bubble sizes correspond to the top three ranked methods for each technology.

**
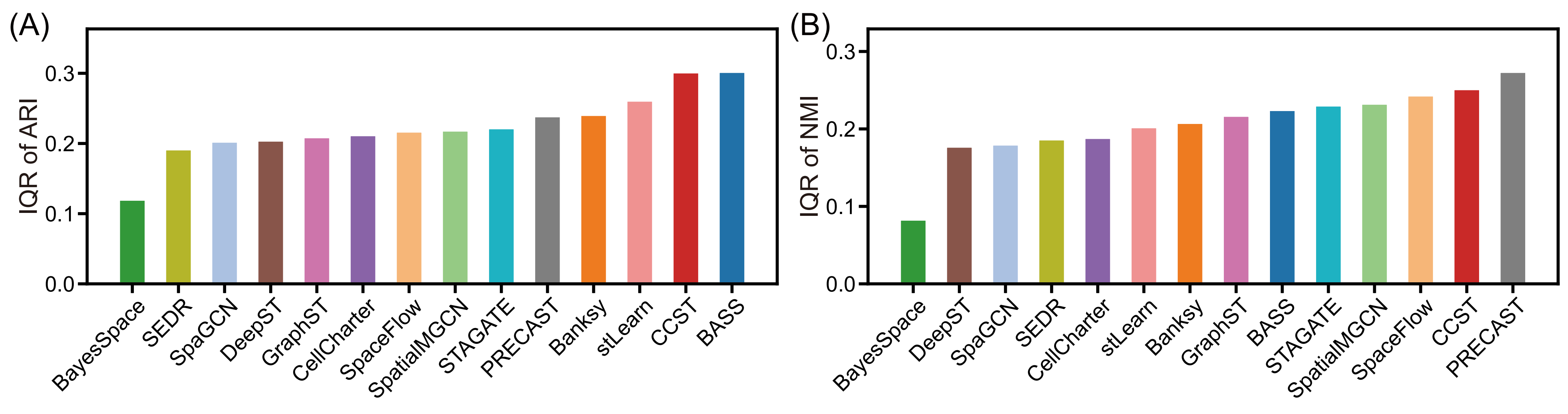
**

**Figure S10** **The stability of spatial clustering methods across all datasets.** The bar plot shows the IQR of the ARI score (A) and the NMI score (B). A smaller IQR indicates more stable performance across technologies. Different colors represent different methods.

**Figure S11** **Computational resource requirements of spatial clustering methods across technologies.** (A) The line plot compares the runtime of each method across datasets from different organs. Line colors indicate methods; shaded areas represent the IQR around the median. (B) The line plot compares the memory used by methods on datasets from different organs. Line colors indicate methods; shaded areas represent the IQR around the median.

**
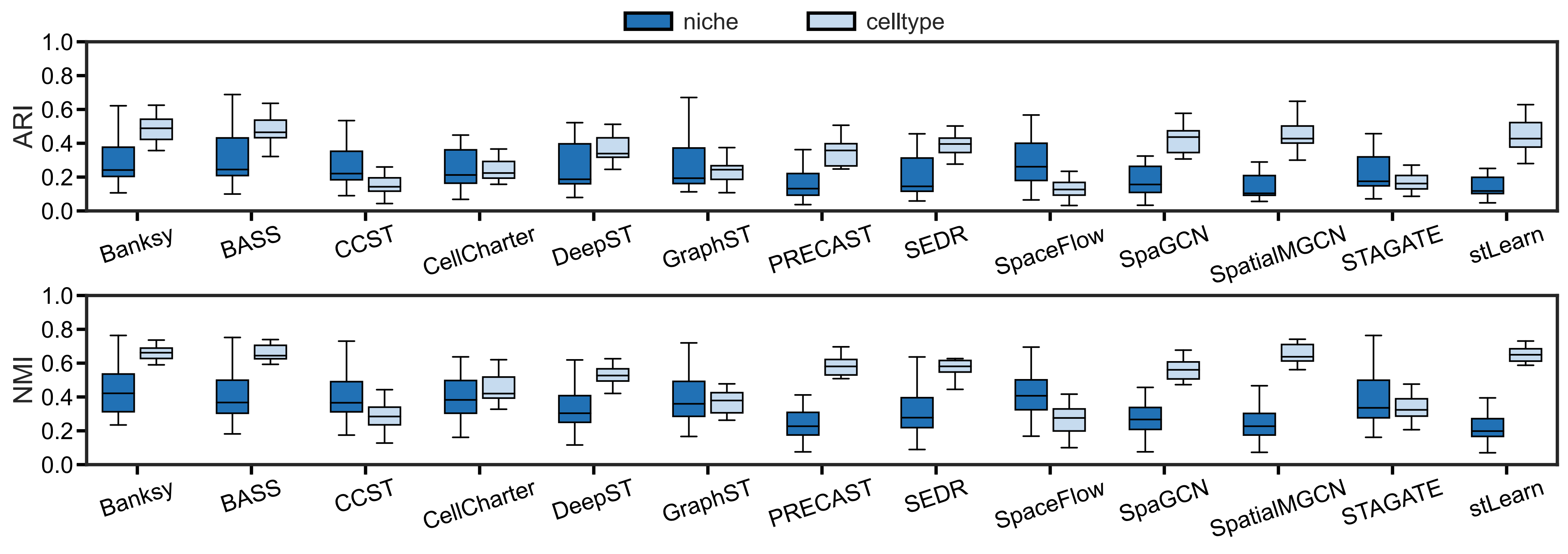
Figure S12** **Performance comparison of methods on cell type clustering and spatial niche detection.** The box plots show clustering accuracy (measured by ARI and NMI) of methods on cell type clustering and spatial domain identification using CosMx datasets. For each metric, the box represents the IQR, the horizontal line inside the box indicates the median, and the whiskers extend to 1.5 × IQR.

**Figure S13** **Performance variation of clustering methods across organs.** (A) Visualization of ground truth annotations and clustering results from all evaluated methods on slice *V19T26-028_D1* from the intestine dataset. The ARI scores are indicated to quantitatively evaluate the accuracy of methods. (B) Visualization of ground truth annotations and clustering results from all evaluated methods on slice *GSM5764414* from the liver dataset. The ARI scores are indicated to quantitatively evaluate the accuracy of methods.

**Figure S14** **Spatial continuity varies across organs in 10× Visium datasets.** The violin plots show the distribution of the spatial continuity metric (PAS, CHAOS, and ASW) across slices from different organs, calculated with ground truth. Each violin represents one organ, and the width indicates the density of values. Colors correspond to organ types.

**Figure S15** **The other accuracy metrics of the methods on the variable organs.** The boxplots of comparing methods on all brain datasets of variable technologies with FMI, Purity, Homogeneity, and Completeness. Center line: median; box limits: upper and lower quartiles; whiskers: 1.5× IQR. Color: technologies.

**
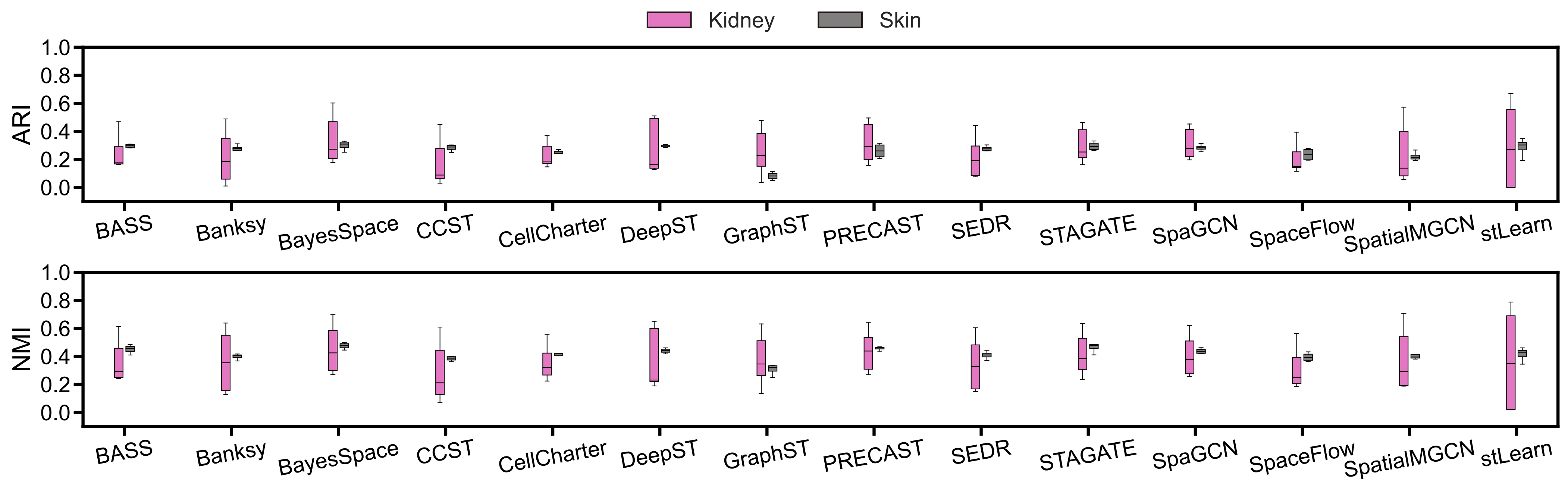
**

**Figure S16** **Performance comparison with SRT datasets from kidney and skin organs.** The box plots compare methods on all 10× Visium datasets of variable organs with ARI and NMI. Centerline: median; box limits: upper and lower quartiles; whiskers: 1.5× IQR. Please pay special attention that the ground truth of these datasets comes from the previous clustering results rather than manual verification.

**Figure S17** **The overall performance of the methods on variable organs.** (A) The bubble plot of accuracy scores for 14 methods on all 10× Visium datasets of variable organs, where a lower accuracy score indicates a higher ranking in terms of method accuracy, and larger bubble sizes correspond to the top three ranked methods for each organ. (B) The bubble plot of continuity scores for 14 methods on all 10× Visium datasets of variable organs, where a lower continuity score indicates a higher ranking, and larger bubble sizes correspond to the top three ranked methods for each organ.

**Figure S18** **The quantitative evaluation metrics of spatial continuity on variable organs.** The boxplots of comparing methods on 10× Visium datasets of variable organs with PAS, CHAOS, and ASW. Center line: median; box limits: upper and lower quartiles; whiskers: 1.5× IQR.


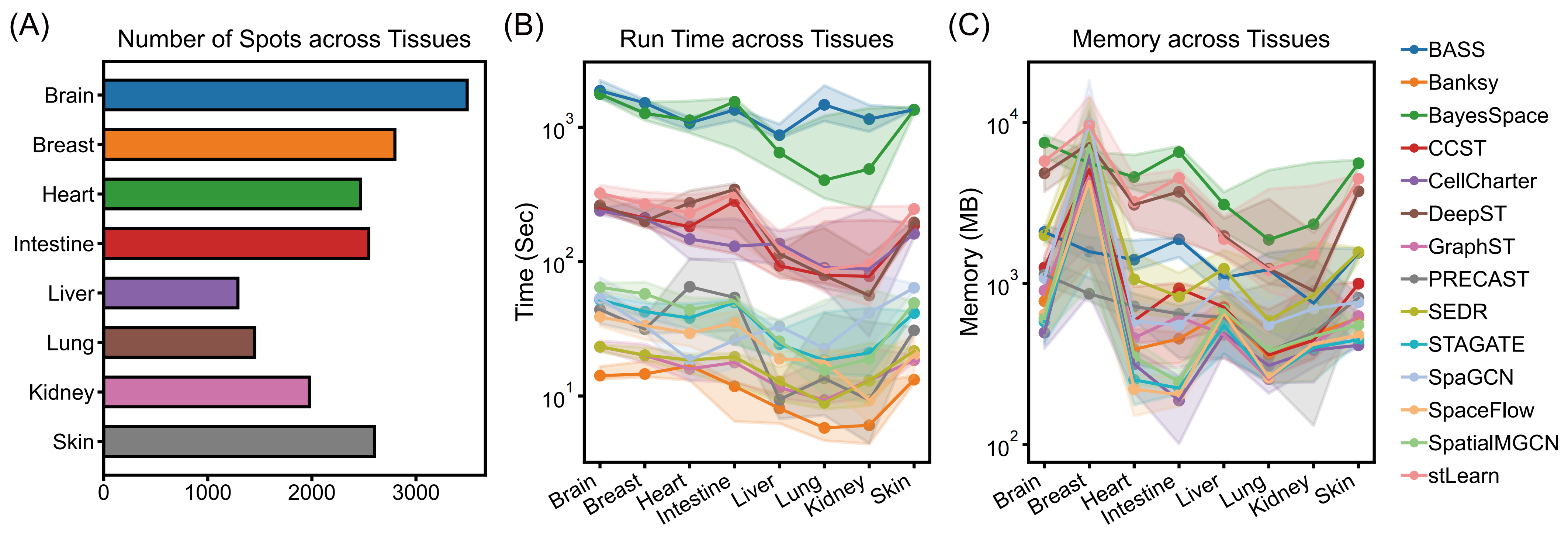


**Figure S19** **Computational resource requirements of spatial clustering methods across organs.** (A) The bar plot shows the average number of spots in datasets across different organs. Colors represent organ types; horizontal bars indicate mean values. (B) The line plot compares the runtime of each method across datasets from different organs. Line colors indicate methods; shaded areas represent the IQR around the median. (C) The line plot compares the memory used by methods on datasets from different organs. Line colors indicate methods; shaded areas represent the IQR around the median.

**Figure S20** **Spatial continuity varies across organs in Slide-seq datasets.** The violin plots show the distribution of the spatial continuity metric (PAS, CHAOS, and ASW) across slices from different organs, calculated with ground truth. Each violin represents one organ, and the width indicates the density of values. Colors correspond to organ types.

**
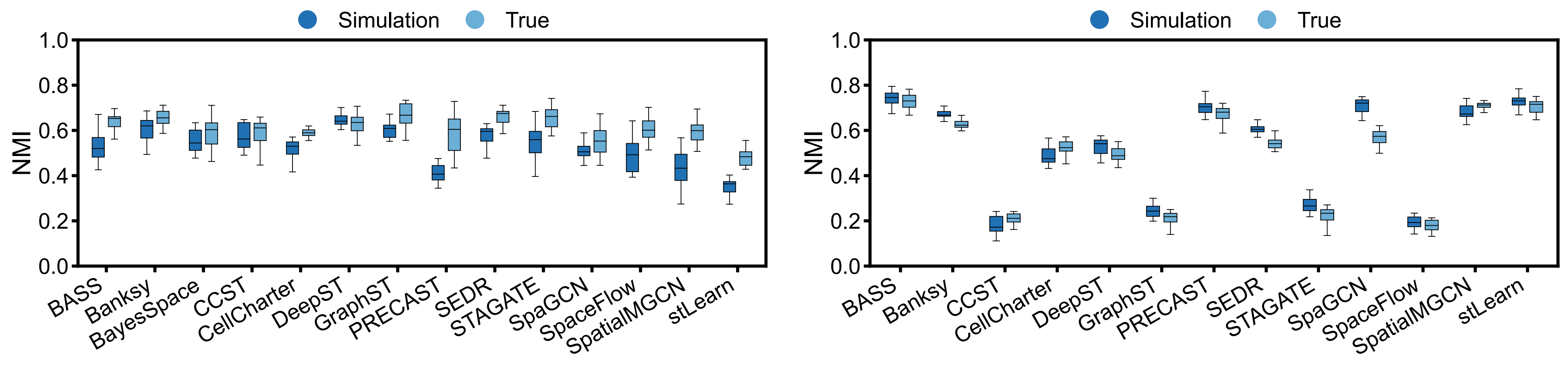
**

**Figure 21 Performance comparison of methods on real datasets and simulated datasets.** The box plots show the NMI score of methods on real datasets and simulated datasets, including 10× Visium and MERFISH separately. For each metric, the box represents the IQR, the horizontal line inside the box indicates the median, and the whiskers extend to 1.5 × IQR.

**Figure S22** **Computational resource requirements of spatial clustering methods across simulated datasets from variable spatial patterns.** The line plots compare the runtime (A) and memory used (B) of each method across datasets from different spot numbers, gene numbers, sparsity, and cluster numbers. Line colors indicate methods; shaded areas represent the IQR around the median.

**
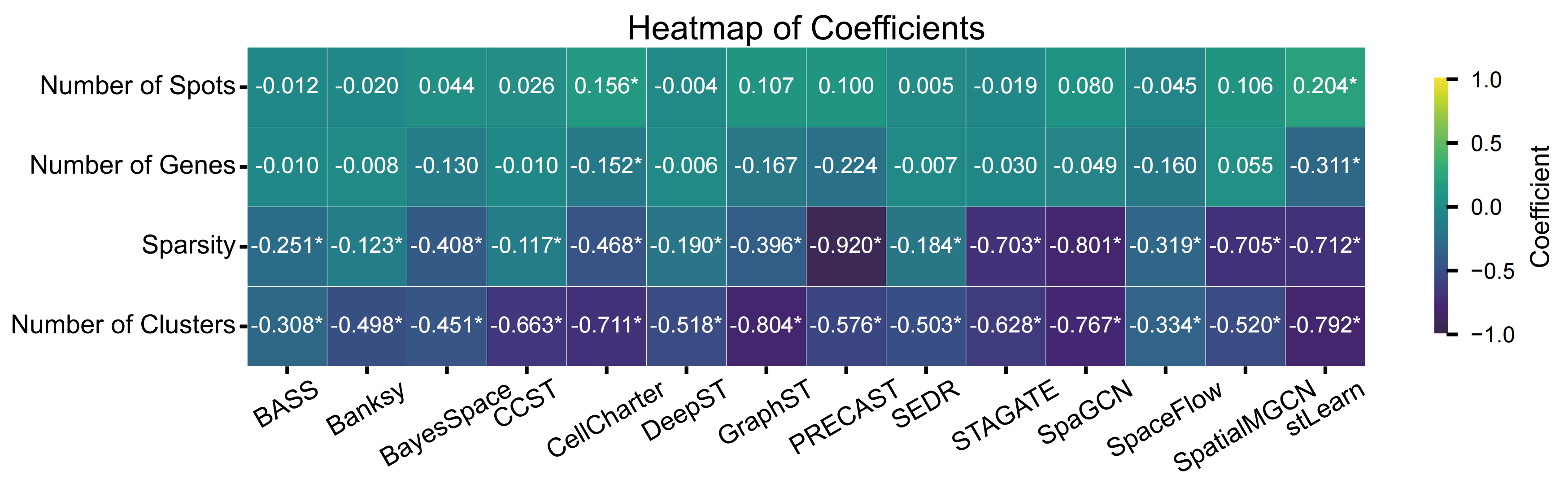
**

**Figure S23** **Contribution of data characteristics to clustering accuracy (ARI).** The heatmap summarizes the estimated coefficients from regression models that quantify the influence of the number of spots, the number of genes, sparsity, and the number of clusters on clustering accuracy measured by ARI. Each cell shows the regression coefficient for a given method and feature, with asterisks indicating statistically significant effects (*p* < 0.05). Positive values suggest a positive association with ARI, while negative values indicate a detrimental impact.

**
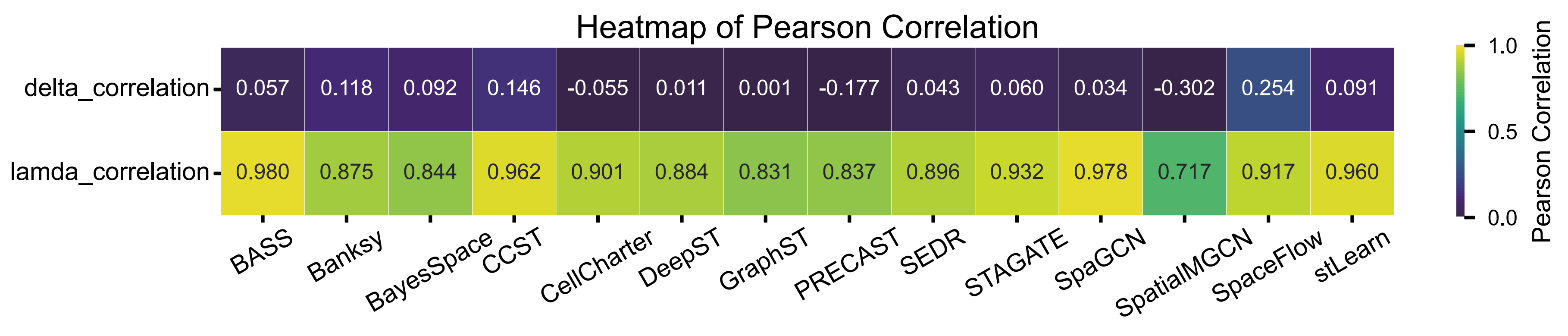
Figure S24** **Pearson correlation of parameters, λ and δ, to ARI.** The heatmap summarizes the Pearson correlation of the parameters, λ and δ, on clustering accuracy measured by ARI.

**Figure S25** **Top 8 preprocessing pipeline of methods (Banksy, BASS, BayesSpace, CCST, CellCharter, DeepST, and GraphST).** For each method, parameter combinations are ranked by ARI score, and the top eight are displayed. Each path represents a parameter combination, while at each step, the parameter most frequently selected across these combinations is shown with a darker node color.

**Figure S26** **Top 8 preprocessing pipeline of methods (PRECAST, SEDR, SpaceFlow, SpaGCN, SpatialMGCN, STAGATE, and stLearn).** For each method, parameter combinations are ranked by ARI score, and the top eight are displayed. Each path represents a parameter combination, while at each step, the parameter most frequently selected across these combinations is shown with a darker node color.

**Figure S27** **Evaluation of preprocessing steps and parameters on clustering accuracy.** (A) The optimal combination of preprocessing parameters for each spatial clustering method. Each colored path represents a method, while node color intensity indicates the most frequently selected parameter in each step across all methods. (B) The heatmap shows the F-values from one-way ANOVA analysis, quantifying the impact of each preprocessing step on the clustering accuracy. Higher F values indicate greater influence. Asterisks (*) denote statistically significant effects (*p* < 0.05). (C) The heatmap shows the relative contribution of each preprocessing step to method performance, quantified by the sum of squared differences.

**
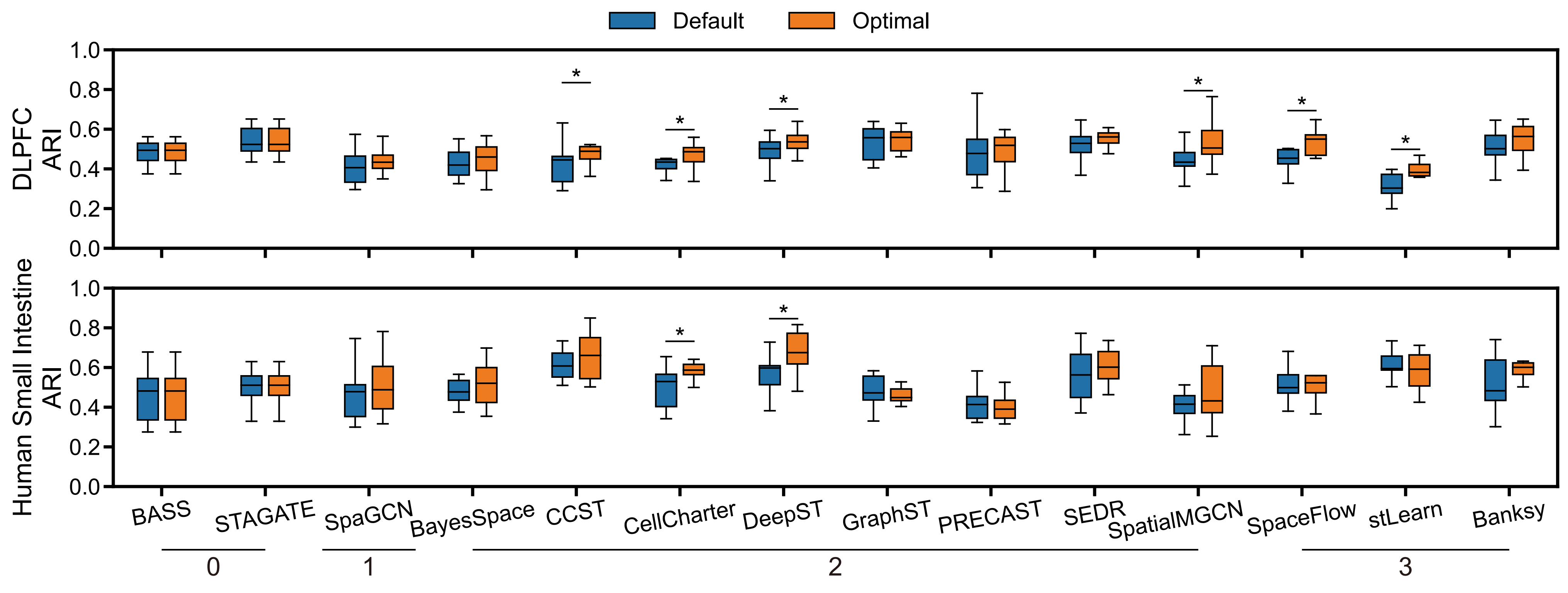
**

**Figure S28 Evaluation of optimal preprocessing pipelines on the DLPFC dataset and the Human Small Intestine dataset.** Boxplots indicate the distribution of ARI scores; the boxes represent the IQR, and whiskers extend to 1.5 × IQR. Significance levels: *p* < 0.05 (*). Numbers indicate the differences between the default and optimized pipelines: 0: identical pipelines; 1: one step differs; 2: two steps differ; and 3: three preprocessing steps differ.

**
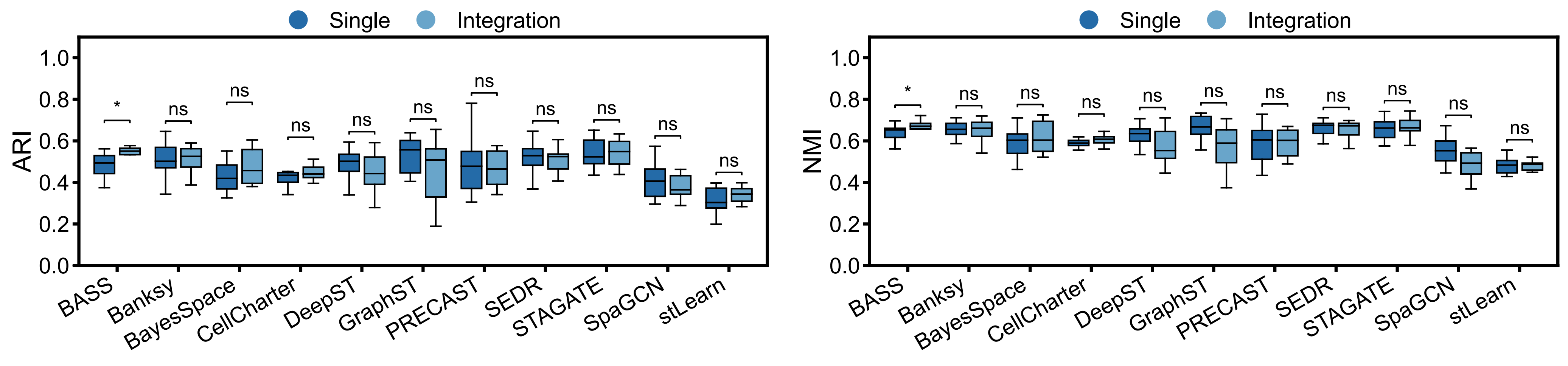
**

**Figure S29** **Comparison of multi-slice integration and single-sample analysis on clustering accuracy.** The box plots show clustering accuracy (measured by ARI and NMI) for multi-slice integration versus single-sample analysis. For each metric, the box represents the IQR, the horizontal line inside the box indicates the median, and the whiskers extend to 1.5 × IQR. Statistical significance is annotated as “ns” (not significant) or “*” (*p* < 0.05).

**Figure S30** **Overall accuracy of clustering methods across datasets.** The heatmap shows the NMI scores of each method across different datasets. The rows represent methods, and the columns represent datasets, annotated with corresponding technology and organ type. The bar plot on the right summarizes the average NMI score of each method across all datasets.

**Figure S31** **Overall accuracy of clustering methods in diverse contexts.** The heatmap shows the NMI scores of each method across different datasets. The rows represent methods, and the columns represent datasets, annotated with variable technologies, organs, and biological replicates.
